# Influence of benzothiophene acceptor moieties on the non-linear optical properties of pyreno-based chromophores: first-principles DFT framework†

**DOI:** 10.1039/d4ra00903g

**Published:** 2024-05-17

**Authors:** Iqra Shafiq, Saadia Haq, Tayyaba Javed, Saifullah Bullo, Sarfraz Ahmed, Norah Alhokbany, Tansir Ahamad

**Affiliations:** a Institute of Chemistry, Khwaja Fareed University of Engineering & Information Technology Rahim Yar Khan 64200 Pakistan; b Centre for Theoretical and Computational Research, Khwaja Fareed University of Engineering & Information Technology Rahim Yar Khan 64200 Pakistan; c Department of Human and Rehabilitation Sciences, Begum Nusrat Bhutto Women University Sukkur Sindh 65170 Pakistan saifullah.bullo@bnbwu.edu.pk; d Wellman Center for Photomedicine, Harvard Medical School, Massachusetts General Hospital Boston MA 02114 USA; e Department of Chemistry, College of Science, King Saud University Riyadh 11451 Saudi Arabia

## Abstract

Herein, a series of heterocyclic organic compounds (PYFD1–PYFD7) are designed with different acceptor moieties at the terminal position of a reference compound (PYFR) for nonlinear optical (NLO) active materials. The optoelectronic characteristics of the designed chromophores were investigated using density functional theory (DFT) calculations with the M06/6-311G(d,p) functional. Frontier molecular orbital (FMO) analysis revealed a significant decrease in the energy of the band gaps (2.340–2.602 eV) for the derivatives as compared to the PYFR reference compound (3.12 eV). An efficient transfer of charge from the highest occupied molecular orbital (HOMO) to the lowest unoccupied molecular orbital (LUMO) was seen, which was further corroborated by the density of states (DOS) and transition density matrix (TDM) heat maps. The results of the global reactivity parameters (GRPs) indicated that all derivatives exhibited greater softness (*σ* = 0.384–0.427 eV) and lower hardness (*η* = 0.394–1.302 eV) as compared to PYFR, indicating a higher level of polarizability in the derivatives. Moreover, all of the derivatives showed significant findings in terms of nonlinear optical (NLO) results as compared to the reference chromophore. PYFD2 showed the most effective NLO response (*α* = 1.861 × 10^−22^ and *β*_tot_ = 2.376 × 10^−28^ esu), including a lowered band gap of 2.340 eV, the maximum softness value of 0.4273 eV, and the lowest hardness value of 1.170 eV as compared to other chromophores. The incorporation of different acceptors and thiophene as a π-spacer in this structural alteration significantly contributed to achieving remarkable NLO responses. Therefore, our findings may motivate experimentalists to synthesize these designed NLO active materials for the current advanced technological applications.

## Abstract 2

Herein, a series of heterocyclic organic compounds (PYFD1–PYFD7) are designed with different acceptor moieties at the terminal position of a reference compound (PYFR) for nonlinear optical (NLO) active materials.

## Graphical abstract

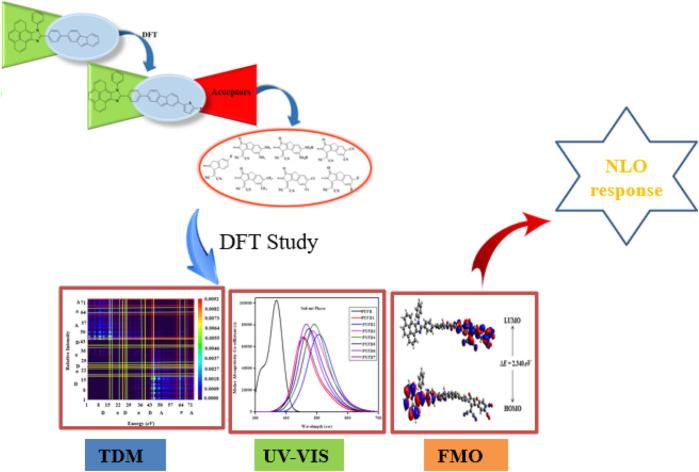

## Introduction

Since the invention of laser light, the remarkable field of non-linear optics has attracted considerable attention owing to their capability to modify the frequency of incident light. Second-order NLO processes are used to apply the NLO effects in a variety of technological domains such as photonic devices,^1^ optical switching, and optical communication.^2^ In recent years, significant efforts have been made in both theoretical and practical endeavors in the advancement of NLO materials.^3,4^ Some instances of NLO devices include frequency converters used in biological imaging technologies and soliton devices, which are used in the massive networked communications.^5,6^

A molecule needs to be stable in at least two states in which it can exhibit very different NLO responses^7^ in order to achieve a significant switching effect. Many scientists have spent years seeking efficient NLO materials including molecular dyes, polymer systems, artificial and synthetic nanoparticles, and inorganic and organic semiconductor diodes.^8,9^ Organic NLO materials possess lower dielectric constant values, higher photoelectrical coefficients, lower development costs, and simpler reaction chemistry. Moreover, their greater ease of use, contribution to frameworks having electron delocalization and greater design freedom make them more favorable than inorganic NLO materials.^10,11^ Centric compounds have a greater capacity for the transfer of charge because donor and acceptor moieties are matched up with organic chromophores that are not based on fullerenes.^12^ Fullerene-free compounds are more flexible in terms of their chemical structure, energy level, electron affinity, and synthesis.

In recent years, a significant number of metal-free organic donor–acceptor complexes have been shown to be exceptional NLO compounds. These complexes exhibit strong intramolecular charge transfer (ICT) properties.^13^ One way that electrons can transport charge inside an electric field is by the movement of electron clouds from the donor to acceptor segments through π-linkers. The ICT development creates a new D–π–A system by generating a “push–pull” interaction between the electron-donating and withdrawing groups. The push–pull configurations can effectively increase the NLO response, which in turn affects their charge separation, band gap, asymmetrical distribution of electrons and the absorption spectra.^14^ Therefore, by using a variety of donors, π-spacers or acceptor moieties, lower band gaps with larger first hyper-polarizability values can be attained for D–π–A systems.^15^ Several systems with an effective push–pull architecture have been published in the literature, which include; D–A, D–π–A, A–D–A, D–A–π–A, D–D–A configurations.^16^

In the present study, we designed a novel family of D–π–A configured, strong push–pull chromophores (PYFD1–PYFD7) by using PYFR (ref. 17) as the reference compound. These compounds are designed by replacing the benzene of PYFR with a highly conjugated system, *i.e*., 2-(7-phenyl-4a*H*-fluoren-2-yl)thiophene (MFT), and then introducing a variety of electron-deficient end-capped acceptor units (FDM, MOM, DTD, DID, ODM, DDM, DOM). According to the literature review, no such study has been reported that describes the NLO properties of PYFR and derived compounds (PYFD1–PYFD7). This report presents the results of an NLO study conducted on the designed D–π–A compounds. To address this research gap, the opto-electronic properties of these newly designed compounds are calculated by utilizing the density functional theory (DFT) and time-dependent DFT (TD-DFT) approach. These chromophores are expected to play a pivotal role in the NLO field. It is believed that this work will inspire the development of metal-free organic molecules with extraordinary NLO capabilities.

## Methodology

Quantum chemical studies of the π-conjugated systems (PYFR and its derivatives, *i.e*., PYFD1–PYFD7) were accomplished using the Gaussian 09 package.^18^ For this purpose, the DFT/TDDFT approaches were utilized at the M06/6-311G(d,p) level.^19,20^ It was found that the above-mentioned level is the highly parameterized approximate new hybrid meta exchange-correlation energy functional of density functional theory (DFT). Moreover, a good harmony was seen between the reported experimental and DFT results at the aforesaid level.^21–24^ Therefore, we are interested in selecting the M06/6-311G(d,p) level for this study. Firstly, the molecular geometries were optimized to obtain their true minima structures at the ground state (S_0_) for the aforesaid level of theory. All of the input files were viewed using the Gauss View 6.0 software.^25^ The time-dependent DFT approach was used to obtain their energy gaps (FMOs and DOS), chemical reactivity (GRPs), and exciton states (TDMs), whereas the hyper-conjugative interactions (NBOs) and non-linear optical (NLO) insights for the said molecules were calculated using the above-mentioned level of DFT. Natural bond orbitals (NBOs) analysis was also carried out. The total dipole moment (*μ*_total_), average linear polarizability (〈*α*〉),^26^ and first (*β*_tot_)^27^ and second hyper-polarizabilities (*γ*_tot_)^28^ were determined using the following eqn (1)–(4).

1 *μ*_total_ = (*μ*_*x*_^2^ + *μ*_*y*_^2^ + *μ*_*z*_^2^)^1/2^

2 〈*α*〉 = 1/3(*a*_*xx*_ + *a*_*yy*_ + *a*_*zz*_)

3 *β*_tot_ = (*β*_*x*_^2^ + *β*_*y*_^2^ + *β*_*z*_^2^)^1/2^

where, *β*_*x*_ = *β*_*xxx*_ + *β*_*xyy*_ + *β*_*xzz*_, *β*_*y*_ = *β*_*yxx*_ + *β*_*yyy*_ + *β*_*yzz*_ and *β*_*z*_ = *β*_*zxx*_ + *β*_*zyy*_ + *β*_*zzz*_.

The dynamic (frequency dependent) first hyperpolarizability is denoted by:

4 *β*(*ω*) = [*β*_*x*_^2^ + *β*_*y*_^2^ + *β*_*z*_^2^]^1/2^

5 *β*_*i*_ = *β*_*ii*_(−2*ω*, *ω*, *ω*) + *β*_*ijj*_(−2*ω*, *ω*, *ω*) + *β*_*ikk*_(−2*ω*, *ω*, *ω*)

for SHG values and

6 *β*_*i*_= *β*_*ii*_(−*ω*, *ω*, 0) + *β*_*ijj*_(−*ω*, *ω*, 0) + *β*_*ikk*_(−*ω*, *ω*, 0)

for EOPE values.7
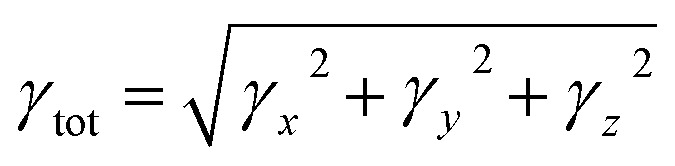
where. 
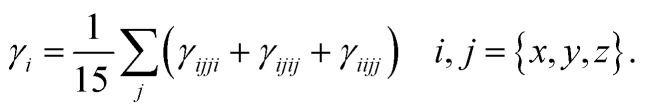


where,

The conclusions were drawn from the output files by using the GaussSum,^29^ Origin 8.0,^30^ Avogadro,^31^ Chemcraft,^32^ Multiwfn 3.8 (ref. 33) and PyMOlyze 2.0 (ref. 34) software programs.

## Results and discussion

**Fig. 1:**
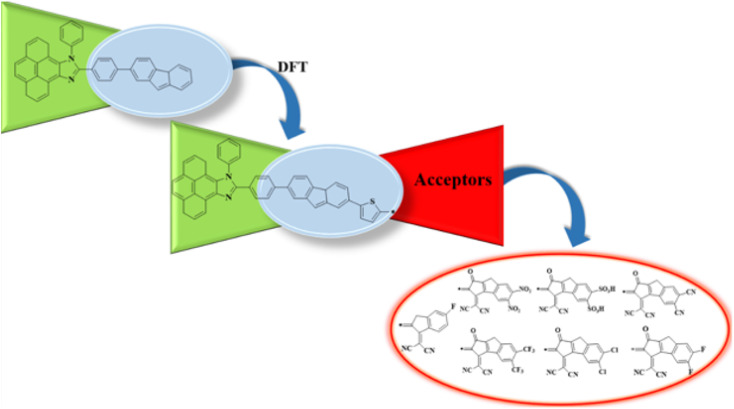
Schematic illustration of the studied compounds (PYFR and PYFD1–PYFD7).

**Fig. 2:**
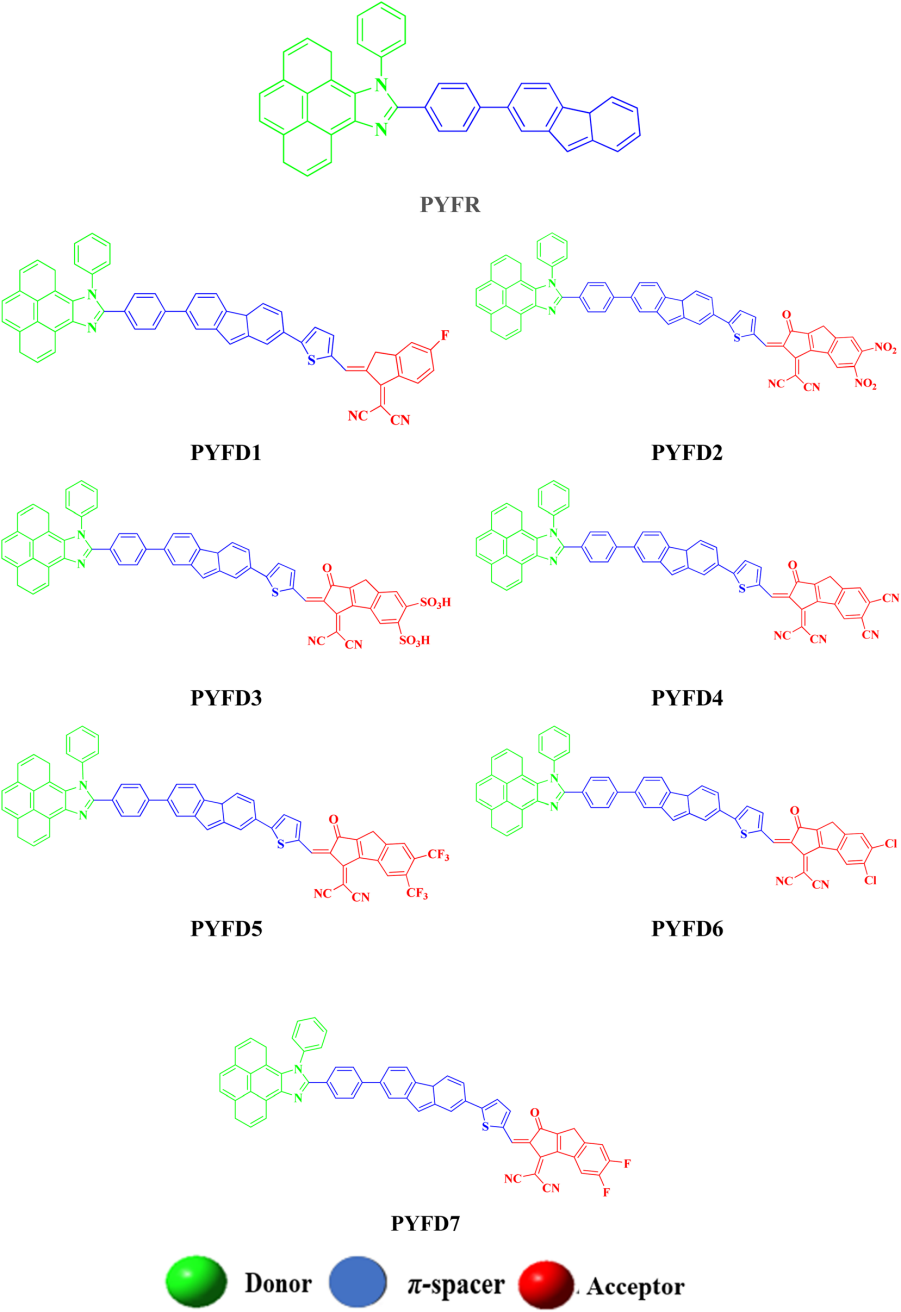
The structures of the investigated chromophores (PYFR and PYFD1–PYFD7).

The current effort focuses on the computational analysis of the NLO response of the designed organic compounds.^35^ Minor changes to the reference molecule (PYFR) led to the design of a series of derivatives (PYFD1–PYFD7), as shown in Fig. 1. Firstly, the π-spacer group of the reference compound is modified by the introduction of 2-methyl-5-(7-(*p*-tolyl)-4a*H*-fluoren-2-yl) thiophene (MFT), which is now considered as the π-linker moiety in the designed chromophores. Further modifications in the derived compounds are made by replacing the previous acceptor group with more efficient acceptor groups, such as 2-(5-fluoro-2-methylene-2,3-dihydro-1-*H*-inden-1-ylidene)malononitrile (FDM), 2-(5,6-dinitro-1-oxo-1,2-dihydrocyclopenta[*a*]inden-3(8-*H*)-ylidene)malononitrile (MOM), 3-(dicyanomethylene)-1-oxo-1,2,3,8-tetrahydrocyclopenta[*a*]indene-5,6-disulfonic acid (DTD), 3-(dicyanomethylene)-2-methylene-1-oxo-1,2,3,8-tetrahydrocyclopenta[*a*]indene-5,6-dicarbonitrile (DID), 2-(2-methylene-1-oxo-5,6-bis(trifluoromethyl)-1,2-dihydrocyclopenta[*a*]inden-3(8-*H*)-ylidene)malononitrile (ODM), 2-(5,6-dichloro-1-oxo-1,2-dihydrocyclopenta[*a*]inden-3(8-*H*)-ylidene)malononitrile (DDM) and 2-(5,6-difluoro-1-oxo-1,2-dihydrocyclopenta[*a*]inden-3(8-*H*)-ylidene)malononitrile (DOM).^36,37^ As a result, seven derivatives (PYFD1–PYFD7) are developed with the modified structural moieties. The optimized structures of PYFR and PYFD1–PYFD7 are displayed in Fig. 2. In this study, the influence of the elongated π-spacer and strongly electron-withdrawing acceptor moieties on the NLO responses, such as the average linear polarizability 〈*α*〉, and first (*β*_tot_) and second hyper-polarizabilities (*γ*_tot_), are investigated. Moreover, several other parameters, such as energy band gaps (*E*_gap_), absorption spectra (UV-Vis), exciton dissociations (*E*_b_), stabilization energies (NBOs) and global reactivity parameters (GRPs): the global softness (*σ*), global electrophilicity index (*ω*), global hardness (*η*), electronegativity (*X*), chemical potential (*μ*), electron affinity (EA) and ionization potential (IP), are also investigated for the designed organic molecules. Cartesian coordinates of the reference and designed compounds are presented in Tables S1–S8† This research study will make a significant contribution to the field of nonlinear optics (NLO) and will inspire other researchers in this field.

### Frontier molecular orbitals (FMOs) analysis

The frontier molecular orbitals (FMOs) theory is considered as a fundamental analysis in predicting the quantum chemical properties of any molecule.^38^ With the help of FMOs, physicists and chemists can describe various aspects of the studied molecules, including their UV-vis spectra, optical properties, electronic features, charge transfer, reactivity and molecular interactions. LUMO stands for the lowest unoccupied molecular orbital and it has a tendency to accept an electron, while HOMO stands for the highest occupied molecular orbital and it has the capacity to donate an electron.^39^ The HOMO–LUMO energy gap (Δ*E*) also provides key evidence related to ICT and NLO behavior.^40,41^ The compounds with high Δ*E* are chemically hard compounds with greater kinetic stability and lower chemical reactivity. In contrast, molecules with a lower *E*_gap_ are softer, more reactive and less stable.^42^ The major findings are shown in Table 1. Meanwhile, energy differences corresponding to HOMO-1/LUMO+1 and HOMO-2/LUMO+2 of the investigated compounds (PYFR and PYFD1–PYFD7) are illustrated in Table S9,† and their corresponding orbitals are shown in Fig. S1.†

**Table 1:** Calculated energies: *E*_HOMO_, *E*_LUMO_, and energy gap (Δ*E*) of the designed compounds in eV

Compounds	*E*_HOMO_	*E*_LUMO_	Δ*E* (eV)
PYFR	−5.455	−2.164	3.291
PYFD1	−5.715	−3.641	2.074
PYFD2	−5.576	−3.958	1.618
PYFD3	−5.577	−3.992	1.585
PYFD4	−5.577	−3.939	1.638
PYFD5	−5.573	−3.869	1.704
PYFD6	−5.580	−3.788	1.792
PYFD7	−5.580	−3.753	1.827

The computed HOMO/LUMO energies of PYFR are −5.455 and −2.164 eV, respectively, with the energy gap of 3.291 eV, as shown in Table 1. The *E*_HOMO_ values were calculated as −5.715, −5.576, −5.577, −5.577, −5.573, −5.580 and −5.580 eV, while the *E*_LUMO_ values were found to be −2.164, −3.641, −3.958, −3.992, −3.939, −3.869, −3.788 and −3.753 eV for PYFD1–PYFD7, respectively. Furthermore, their corresponding *E*_gap_ values were observed as 3.291, 2.074, 1.618, 1.585, 1.638, 1.704, 1.792 and 1.827 eV, respectively. The derivatives exhibited smaller bandgaps than PYFR due to the introduction of various extended acceptors and the addition of the electronic π-bridge (2-methyl-5-(7-phenyl-4a*H*-fluoren-2-yl)thiophene), which results in resonance and the promotion of the transfer of electron density in the D–π–A configured compounds.

All derivatives include strong electron-accepting substituents in their structures; hence, they all exhibit smaller *E*_gap_ values than PYFR, which lie in the range of 2.074–1.585 eV. PYFD2 shows the shortest *E*_gap_ (1.618 eV) compared to all other chromophores due to the presence of a nitro (–NO_2_) group, which has a strong electron-withdrawing property and may drive the electron density away from nearby atoms in a molecule. On the other hand, PYFD1 exhibits the largest band gap (2.074 eV), owing to the presence of the 2-(5-fluoro-2-methylene-2,3-dihydro-1-*H*-inden-1-ylidene)malononitrile acceptor moiety (FDM). Due to the existence of the sulphonic acid (–SO_3_H) group, PYFD3 has shown a smaller band gap than PYFD2 (1.585 < 1.618 eV). Furthermore, in the PYFD4 molecule, the –SO_3_H group is replaced with a –CN group; hence, it depicted a higher band gap of 1.638 eV. In contrast to PYFD5 (1.704 eV), which contains the trifluoromethyl groups (–CF_3_) on its terminal acceptor, PYFD6 and PYFD7 possess –Cl and –F groups linked to their respective benzene rings, and showed enhanced energy gaps as 1.792 and 1.827 eV, respectively. In general, the energy gap trend is summarized as follows: PYFR > PYFD1 > PYFD7 > PYFD6 > PYFD5 > PYFD4 > PYFD3 > PYFD2. Owing to the addition of the highly electron-withdrawing –NO_2_ group in the acceptor moiety along with the modified π-linker in PYFD2, the rate of intramolecular charge transfer (ICT) is primarily enhanced, which has led to a decreased band gap as compared to all other derivatives. The molecular orbital energies and their band gaps calculated *via* DFT approach are a little bit higher than that of TD-DFT (Table S41†).

**Fig. 3:**
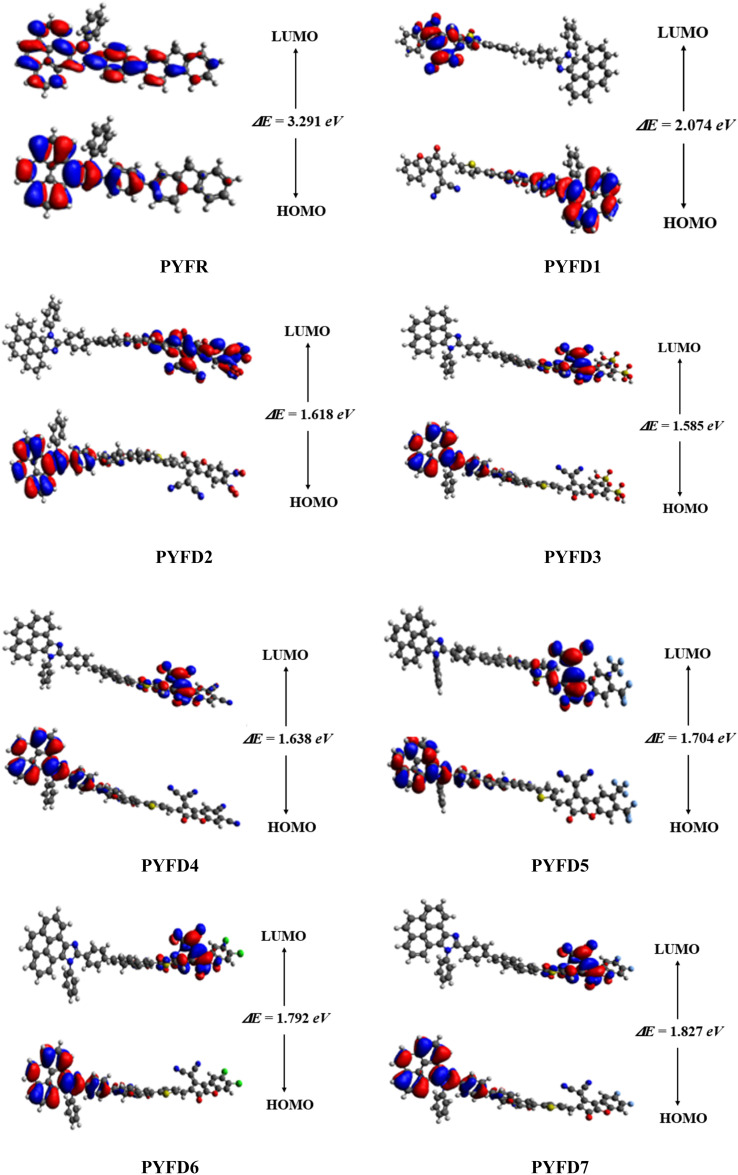
The HOMO/LUMO and their energy gaps of the designed compounds (PYFR and PYFD1–PYFD7).

Fig. 3 illustrates the counter surface diagrams of the FMOs, depicting the electron density distribution over different areas of the molecules. The reference compound (PYFR) displays a unique electronic distribution pattern, with its LUMO entirely covered with the electronic clouds. Conversely, in its HOMO, the electronic clouds are predominantly concentrated in the donor region and the π-spacer. For derivatives (PYFD1–PYFD7), the HOMOs exhibit electron density on their donor regions and partially over the π-spacer. Whereas, in their LUMOs, the acceptor part displays prominent electronic clouds and negligibly over the π-spacer region.

### Global reactivity parameters (GRPs)

The energy gap (*E*_LUMO_–*E*_HOMO_) is the primary determinant for assessing the global reactivity parameters (GRPs) *via* the application of Koopmans' theorem. GRPs include the global softness (*σ*), global electrophilicity index (*ω*), global hardness (*η*), electronegativity (*X*), chemical potential (*μ*), electron affinity (EA) and ionization potential (IP),^43,44^ which are determined by using eqn (8)–(15), and the calculated values are presented in Table 2.

8 IP = −*E*_HOMO_

9 EA = −*E*_LUMO_

10 *X* = (IP + EA)/2]

11 *η* = (IP − EA)

12
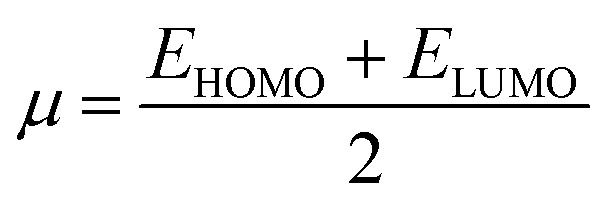
13
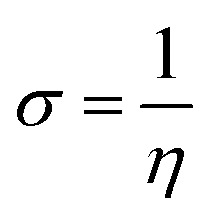
14
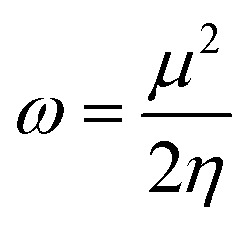

15 Δ*N*_max_ = −*μ*/*η*

**Table 2:** The calculated GRPs of PYFR and MTRID1–MTRID7^a^

Compounds	IP	EA	*X*	*η*	*μ*	*ω*	*σ*	Δ*N*_max_
PYFR	5.735	1.723	3.729	2.006	−3.729	3.4659	0.2492	1.8589
PYFD1	5.729	3.125	4.427	1.302	−4.427	7.5262	0.3840	3.4001
PYFD2	5.739	3.399	4.569	1.170	−4.569	8.9212	0.4273	3.9051
PYFD3	5.740	3.391	4.5655	1.174	−4.565	8.8734	0.4257	3.8871
PYFD4	5.738	3.350	4.5440	1.194	−4.5440	8.6465	0.4187	3.8056
PYFD5	5.735	3.285	4.5100	1.225	−4.5100	8.3020	0.4081	3.6816
PYFD6	5.731	3.220	4.4755	1.255	−4.4755	7.9769	0.3982	3.5647
PYFD7	5.733	3.198	4.4655	0.394	−4.4655	7.8661	0.3944	3.5230

a Units in eV. Global softness in eV^−1^.

The energy needed to remove an electron from the highest occupied molecular orbital (HOMO) is equal to the ionization potential (IP), which is used to determine the electron-donating and electron-accepting capabilities of an atom.^45^ In our studied compounds (PYFR and PYFD1–PYFD7), the ionization potential (IP) values existed within the range of 5.729–5.740 eV. A similar parameter is known as electron affinity (EA), which denotes the electron accepting tendency of chromophores. The statistical values obtained for our designed molecules (PYFD1–PYFD7) are higher than the reference compound (PYFR), which is due to their higher tendency to receive electrons owing to the presence of strong acceptor groups. The decreasing order of EA of the studied compounds in eV is as follows: PYFD2 (3.399) > PYFD3 (3.391) > PYFD4 (3.350) > PYFD5 (3.285) > PYFD6 (3.220) > PYFD7 (3.198) > PYFD1 (3.125) > PYFR (1.723). Electronegativity (*X*) is a chemical property which quantifies the attraction of an atom towards the electrons in a chemical bond. The chemical potential (*μ*) of a species facilitates comprehension of a compound's stability and reactivity. These metrics quantify the electrophilic strength of a compound. Moreover, there is a direct relationship of the chemical stability with the energy gap, chemical potential and global hardness of an organic compound, while they are inversely related to the reactivity and softness of a compound. Therefore, the softer molecules have a smaller bandgap, which makes them more reactive. The polarizability of molecules may be linked with their softness, since softer molecules are often more polarized. Among all the designed compounds, PYFD2 showed the highest value of softness (*σ*), *i.e*., 0.4273 eV^−1^, which demonstrated the highest polarizability and increased reactivity. The extent of softness (*σ*) was decreased to 0.4257 eV^−1^ in PYFD3. A decrease in the value is seen in the case of PYFD4, PYFD5, PYFD6 and PYFD7, with values at 0.4187, 0.4081, 0.3982 and 0.3944 eV^−1^, respectively. The lowest *σ* value, indicating minimal reactivity and lower polarizability, was found to be in PYFD1 as 0.3840 eV^−1^. The observed order of global softness (*σ*) is as follows: PYFD2 > PYFD3 > PYFD4 > PYFD5 > MTRID6 > PYFD7 > PYFD1 > PYFR. Similarly, in the case of its counter parameter, which is known as the global hardness (*η*), out of all the compounds, PYFR exhibited the highest value (2.006 eV), while PYFD7 was found to have the lowest value at 0.394 eV.

It is worth noting that all the chromophores exhibited greater global softness (0.3840–0.4273 eV^−1^) and lower hardness values (0.394–1.302 eV). The elevated levels of softness observed suggest the enhanced reactive nature of the investigated compounds. Overall, this study revealed that the molecules' exhibited efficient CT ability between their HOMOs and LUMOs leads to better polarizability and remarkable NLO behavior.^46^

### Density of states (DOS)

**Fig. 4:**
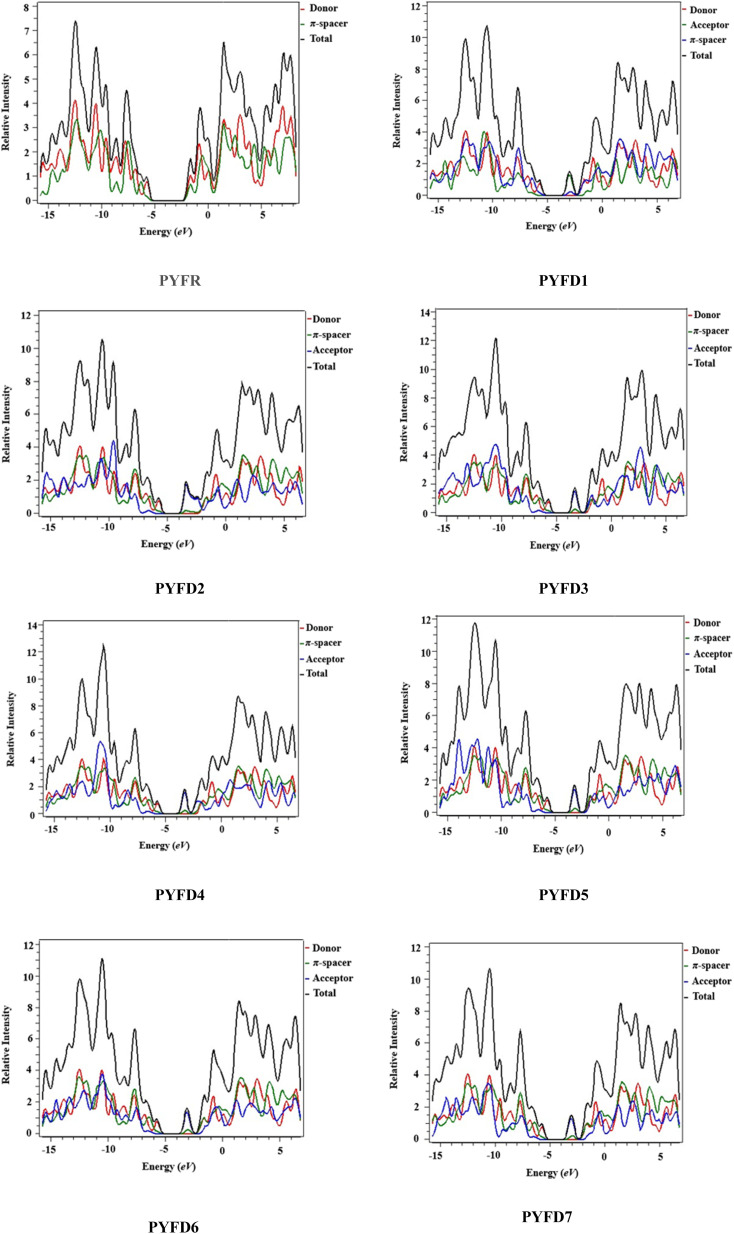
DOS pictographs of PYFR and PYFD1–PYFD7.

Density of states (DOS) plots play a crucial role in examining the distribution and positioning of HOMO and LUMO densities.^16^ These plots are employed to investigate the correlation between chemical bonding and the overlap population of molecules with various acceptor–donor combinations.^47^ The present DOS study is conducted for PYFR and PYFD1–PYFD7 and the resulting graphical plots are displayed in Fig. 4. These visualizations offer detailed insight into the energy levels of the frontier orbitals and the electronic states within the molecules. Such information is fundamental for predicting the electronic and optical properties of the studied molecules. In conducting DOS investigations, the compounds under study are broken down into donor, π-core and end-capped acceptor units, each denoted by a distinct color (donor in red, π-spacer in green and acceptor in blue lines) in PYFD1–PYFD7. The alteration in the pattern of the charge distribution is influenced by changes in the acceptor moieties, and is substantiated by the HOMO–LUMO percentages of DOS displayed in Table S10.†

The data shown in the table depicts the percentages for all possible fragments of our studied chromophores. In the case of the reference molecule (PYFR), the donor shows a high percentage on the HOMO (83.4%), while there is 61.3% on the LUMO. Contrary to this, its π-spacer shows a greater contribution towards the LUMO as 38.7% and less contribution towards the HOMO (16.6%). Moreover, the acceptor moiety is absent in PYFR. For PYFD1–PYFD7, the maximum charge on HOMOs is located over the donor moieties, *i.e.*, 83.1, 83.7, 83.8, 83.6, 83.5, 83.2 and 83.3%, correspondingly. While, in the case of LUMOs, the highest DOS percentages are recorded for acceptors such as 92.1, 80.8, 91.1, 91.6, 90.7, 91.6 and 92.3% in PYFD1–PYFD7, correspondingly. The π-spacer functions as a facilitator with intermediate charge contributions for HOMOs and LUMOs. However, slightly greater contributions of π-spacers are recorded for their HOMOs as 16.9, 16.2, 16.1, 16.3, 16.5, 16.8 and 16.6% for PYFD1–PYFD7, respectively. The positive region on the DOS graphs indicates the LUMOs peaks and the negative regions displayed corresponding peaks for HOMOs. The energy gap between HOMOs and LUMOs of the respective compounds can be marked *via* the distance between their foremost HOMO/LUMO peaks from the graph. It can be depicted feasibly from the DOS plot that in the LUMO region, the blue peaks are highest, which supports the high acceptor participation in the LUMOs from Table S11.† Similarly, the HOMOs region showed dominant donor peaks (red colored).

The DOS analysis of our concerned molecules firmly supports their frontier orbitals studies. Moreover, it reveals the efficient delocalization of the electronic charge from the electron-rich donor towards the electron-deficient acceptor, which is consistent in all of the designed compounds.

### Natural bond orbitals (NBOs) analysis

NBOs analysis provides a comprehensive understanding of the intra- and intermolecular interactions between the donor and acceptor components, as well as insights into the conjugative interactions and charge transfer within the molecules.^48^ The stabilization energy *E*^(2)^ associated with the donor–acceptor interactions can be effectively determined using the second-order perturbation theory. In NBOs analysis, the donor (*i*) transfers the charge density to the acceptor (*j*) *via* the π-linker, facilitating the transfer of electronic charges.^49^ The delocalization of charges and the corresponding stabilization energies (*E*^(2)^) of our investigated molecules (PYFR and PYFD1–PYFD7) can be calculated using eqn (16). The donor orbital occupancy (q_*i*_), off-diagonal elements (*ε*_*i*_ and *ε*_*j*_), and diagonal NBO Fock matrix elements (*F*_*i.j*_) are used to calculate the stabilization energy (*E*^(2)^) and can be expressed as follows:16
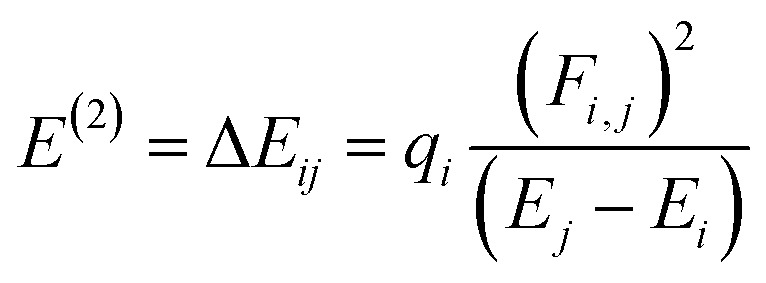


A higher *E*^(2)^ indicates a more pronounced interaction between the electron donors, signifying increased conjugation through the entire system. Utilizing the same DFT functional, the NBOs analysis for the designed molecules is conducted, and the essential transitions are summarized in Table 3. ESI† regarding the interactions can be found in Tables S11 through S18,† providing additional details on the analysis.

**Table 3:** Nominated values of NBOs analysis for PYFR and PYFD1–PYFD7

Compounds	Donor (*i*)	Type	Acceptor (*j*)	Type	*E*(*j*)*E*(*i*)	*F*(*i*,*j*)	*E*(*j*)*E*(*i*)
PYFR	C9–N37	σ	C25–C26	σ*	6.74	1.28	0.083
	C24–N36	σ	C25–N37	σ*	0.50	1.35	0.023
	C65–C67	π	C60–C62	π*	23.98	0.29	0.075
	C60–C62	π	C28–C31	π*	0.60	0.32	0.012
	N36	LP(1)	C25–N37	π*	51.64	0.30	0.111
	N37	LP(1)	C25–N36	σ*	10.08	0.81	0.081
PYFD1	C69–H70	σ	C71–C72	σ*	9.40	1.00	0.086
	C47–H49	σ	C47–C51	σ*	0.50	0.95	0.020
	C81–C82	π	C78–C80	π*	25.50	0.28	0.078
	C89–N90	π	C91–N92	π*	0.64	0.47	0.016
	N36	LP(1)	C25–N37	π*	51.69	0.30	0.111
	N90	LP(1)	C75–C89	σ*	12.69	1.04	0.103
PYFD2	C69–H70	σ	C71–C72	σ*	9.39	1.00	0.087
	C81–C82	σ	C82–N100	σ*	0.50	1.02	0.021
	C38–C39	π	C44–C46	π*	24.73	0.30	0.078
	C89–N90	π	C87–N88	π*	0.60	0.48	0.015
	N36	LP(1)	C25–N37	π*	51.77	0.30	0.111
	O91	LP(2)	C73–C76	σ*	24.22	0.73	0.120
PYFD3	C69–H70	σ	C71–C72	σ*	9.33	1.00	0.086
	O77–C80	σ	C76–O77	σ*	0.51	1.31	0.023
	C38–C39	π	C44–C46	π*	24.75	0.30	0.078
	C89–N90	π	C87–N88	π*	0.59	0.48	0.015
	N36	LP(1)	C25–N37	π*	51.75	0.30	0.111
	O91	LP(2)	C71–C73	σ*	21.68	0.71	0.113
PYFD4	C69–H70	σ	C71–C72	σ*	9.44	1.00	0.087
	C47–H49	σ	C47–C51	σ*	0.50	0.95	0.020
	C38–C39	π	C44–C46	π*	24.72	0.30	0.078
	C89–N90	π	C87–N88	π*	0.61	0.48	0.015
	N36	LP(1)	C25–N37	π*	51.79	0.30	0.111
	O91	LP(2)	C73–C76	σ*	24.11	0.73	0.12
PYFD5	C69–H70	σ	C71–C72	σ*	9.48	1.00	0.087
	C47–H49	σ	C47–C51	σ*	0.50	0.95	0.020
	C38–C39	π	C44–C46	π*	24.70	0.30	0.078
	C89–N90	π	C87–N88	π*	0.61	0.48	0.015
	O77	LP(2)	C74–C76	π*	34.67	0.39	0.104
	O91	LP(2)	C73–C76	σ*	23.86	0.73	0.119
PYFD6	C69–H70	σ	C71–C72	σ*	9.50	1	0.087
	C81–H83	σ	C82–Cl100	σ*	0.50	0.68	0.017
	C38–C39	π	C44–C46	π*	24.67	0.30	0.078
	C89–N90	π	C87–N88	π*	0.62	0.48	0.015
	N36	LP(1)	C25–N37	π*	51.78	0.30	0.111
	O91	LP(2)	C73–C76	σ*	23.55	0.73	0.119
PYFD7	C69–H70	σ	C71–C72	σ*	9.35	1	0.086
	C47–H49	σ	C47–C51	σ*	0.50	0.95	0.020
	C38–C39	π	C44–C46	π*	24.65	0.30	0.078
	C93–C96	π	C55–C56	π*	11.42	0.32	0.056
	N36	LP(1)	C25–N37	π*	51.69	0.30	0.111
	O91	LP(2)	C73–C76	σ*	23.46	0.74	0.119

Generally, the possible electronic transitions are as follows: σ → σ*, π → π*, LP → σ* and LP → π*. Among above mentioned transitions, π → π* transitions are considered to be predominant, which occur due to the π-conjugation system, σ → σ* are weaker due to sigma bonds, and LP → σ* and LP → π* are minutely prominent excitations. According to Table 1, the significant π → π* transitions for PYFR and PYFD1–PYFD7 are as follows: π(C65–C67) → π*(C60–C62), π(C81–C82) → π*(C78–C80), π(C38–C39) → π*(C44–C46), π(C38–C39) → π*(C44–C46), π(C38–C39) → π*(C44–C46), π(C38–C39) → π*(C44–C46), π(C38–C39) → π*(C44–C46) and π(C38–C39) → π*(C44–C46) with stabilization energies as 23.98, 25.50, 24.73, 24.75, 24.72, 24.7, 24.67 and 24.65 kcal mol^−1^, respectively. However, the lowest energy π → π* transitions are characterized as π(C60–C62) → π*(C28–C31), π(C89–N90) → π*(C91–N92), π(C93–C96) → π*(C93–C96), π(C93–C96) → π*(C93–C96), π(C93–C96) → π*(C95–C96), π(C93–C96) → π*(C95–C96), π(C89–N90) → π*(C87–N88) and π(C81–C82) → π*(C81–C82), with the associated values as 0.60, 0.64, 0.58, 0.58, 0.58, 0.54,0.62 and 0.55 kcal mol^−1^ for the PYFR and PYFD1–PYFD7 compounds, respectively.

In σ → σ* transitions, the highest energy of stabilization are obtained to be 6.74, 9.40, 9.39, 9.33, 9.44, 9.48, 9.50 and 9.35 kcal mol^−1^ for σ(C9–N37) → σ*(C25–S26), σ(C69–H70) → σ*(C71–C72), σ(C69–H70) → σ*(C71–C72), σ(C69–H70) → σ*(C71–C72), σ(C69–H70) → σ*(C71–C72), σ(C69–H70) → σ*(C71–C72), σ(C69–H70) → σ*(C71–C72) and σ(C69–H70) → σ*(C71–C72) transitions in PYFR and PYFD1–PYFD7, correspondingly. On the other hand, the lowest σ → σ* transition energy values are also recorded for the above-mentioned compounds as 0.50, 0.50, 0.50, 0.51, 0.50, 0.50, 0.50 and 0.51 kcal mol^−1^, respectively, for the σ(C24–N36) → σ*(C25–N37), σ(C47–H49) → σ*(C47–C51), σ(C81–C82) → σ*(C82–N100), σ(O77–C80) → σ*(C76–O77), σ(C47–H49) → σ*(C47–C51), σ(C47–H49) → σ*(C47–C51), σ(C81–H83) → σ*(C82–Cl100) and σ(C47–H49) → σ*(C47–C51) transitions.

The LP → π* transitions, *i.e.*, LP1(N36) → π*(C25–N37), LP1(N36) → π*(C25–N37), LP1(N36) → π*(C25–N37), LP1(N36) → π*(C25–N37), LP1(N36) → π*(C25–N37), LP2(O77) → π*(C74–C76), LP1(N36) → π*(C25–N37) and LP1(N36) → π*(C25–N37), demonstrate significant stabilization energies as 51.64, 51.69, 51.77, 51.75, 51.79, 34.67, 51.78 and 51.69 kcal mol^−1^ for PYFR and PYFD1–PYFD7, correspondingly. The LP → σ* transitions, LP1(N37) → σ*(C25–N36), LP1(N90) → σ*(C75–C89), LP2(O91) → σ*(C73–C76), LP2(O91) → σ*(C71–C73), LP2(O91) → σ*(C73–C76), LP2(O91) → σ*(C73–C76), LP2(O91) → σ*(C73–C76) and LP2(O91) → σ*(C73–C76), showed the smallest transition energy values as 10.08, 12.69, 24.22, 21.68, 24.11, 23.86, 23.55 and 23.46 kcal mol^−1^, correspondingly, for PYFR and PYFD1–PYFD7.

The above-mentioned results showed that PYFD1 exhibited the extra stability (25.50 kcal mol^−1^) among the studied compounds. This is due to its prolonged hyper-conjugative interactions. Overall, the NBOs study revealed that the investigated chromophores are stabilized due to hyper-conjugation, which plays a key role in their better NLO responses.

### Transition density matrix (TDM)

**Fig. 5:**
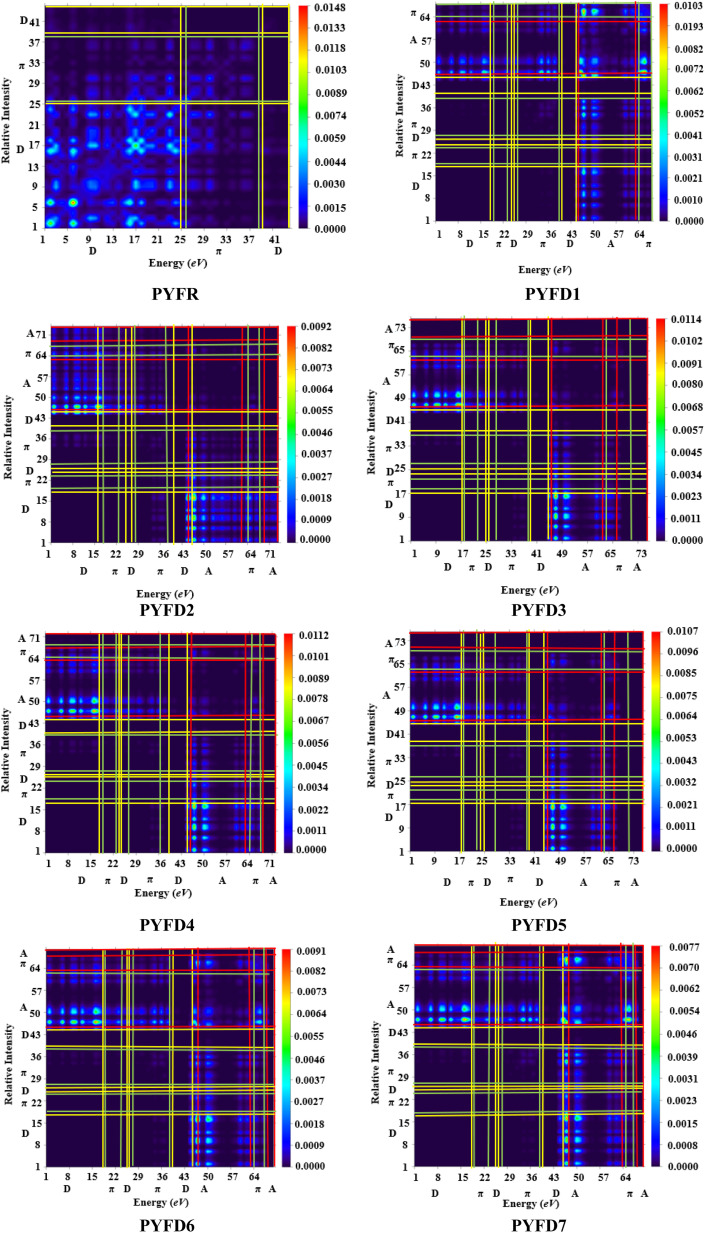
TDM heat maps of the investigated molecules PYFR and PYFD1–PYFD7.

The transition density matrix (TDM) is a valuable tool used for determining the electronic charge transfer in the designed compounds (PYFD1–PYFD7) and the reference molecule (PYFR). The TDM is used to assess the correlation between the donor and acceptor parts in the excited state, as well as the localization and delocalization of electron–hole pairs.^50^ It is often used to comprehensively analyze the magnitude and characteristics of transitions in the compounds under investigation.^51^ This study excludes the impact of the hydrogen atom since it makes a negligible contribution to electronic transitions. The TDM findings for all of the developed compounds are shown in Fig. 5. In order to comprehend the transfer of charge density, we subdivided our reference compound (PYFR) into two components: a donor and a π-linker. The fabricated chromophores (PYFD1–PYFD7) are divided into three segments, namely D, π-linker, and A. The TDM maps exhibited a very efficient diagonal charge transfer (CT) coherence in all of the chromophores. In PYFR, the majority of the charge density is concentrated in the donor region. However, for all of the designed compounds, the majority of the electron density is located on the acceptor region. Only a small portion of the electron density is observed in the π-linker, as indicated by the green spots. These green spots demonstrate the successful transfer of electron coherence from the donor to the π-linker, which effectively facilitates the movement of electron density towards the acceptor without any trapping. The results from the TDM heat maps suggested that there is a well-defined and enhanced separation of excitons in the excited state, which is crucial for the advancement of the NLO material.

### Binding energy (*E*_b_)

The binding energy is a crucial and promising factor for determining the optoelectronic characteristics. It aids in identifying the possibility for excitation dissociation. Decreasing the binding energy reduces the strength of the coulombic interactions between the hole and electron, resulting in improved exciton dissociation in the excited state.^52^ It is determined by subtracting the HOMO–LUMO energy gap from the first exciton energy. The binding energy (*E*_b_) values of the PYFR and PYFD1–PYFD7 compounds were determined using eqn (17).

17 *E*_b_ = *E*_L−H_ − *E*_opt_

The symbol *E*_b_ represents the binding energy, *E*_L-H_ indicates the bandgap and *E*_opt_ denotes the initial excitation energy. The calculated results for the binding energy are shown in Table 4.

**Table 4:** Calculated binding energy (*E*_b_) of the designed compounds in eV

Compounds	Δ*E*	*E*_opt_	*E*_b_
PYFR	4.012	3.342	0.670
PYFD1	2.604	2.239	0.365
PYFD2	2.340	1.807	0.533
PYFD3	2.349	1.778	0.571
PYFD4	2.388	1.847	0.541
PYFD5	2.450	1.969	0.481
PYFD6	2.511	2.095	0.416
PYFD7	2.535	2.105	0.430

The data presented above indicate that all of the chromophores had lower binding energy values (0.365 to 0.571 eV) compared to the reference chromophore (0.670 eV). This reduced *E*_b_ can be attributed to the alteration in their structure, resulting in a robust push–pull configuration. The lower excitation and band gap energy levels result in a decreased binding energy, which in turn facilitates greater excitation dissociation and higher charge mobility rate. This leads to superior optoelectronic characteristics. The binding energy values decrease in the following order: PYFR > PYFD3 > PYFD4 > PYFD2 > PYFD5 > PYFD7 > PYFD6 > PYFD1. Molecules with low binding energy values have a strong correlation with polarizability. From previous studies, it was observed that the molecules with *E*_b_ values below 1.9 eV are regarded as excellent photonic materials, which exhibit a notable NLO response. Notably, all of our derivatives exhibited a binding energy below 1.9 eV, indicating their potential as NLO materials due to their substantial charge separation in excited states.

### UV-vis analysis

**Fig. 6:**
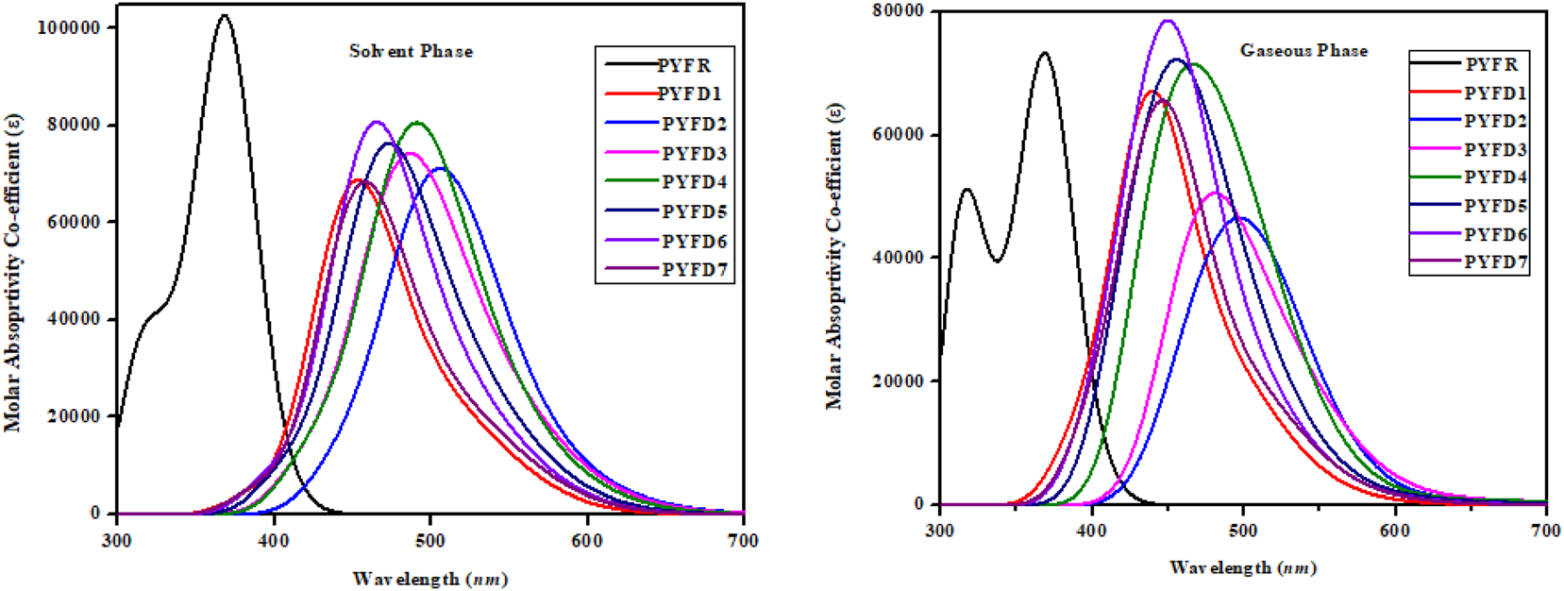
UV-vis absorption spectra of the investigated molecules, PYFR and PYFD1–PYFD7.

The UV-Vis spectroscopic technique was conducted to elucidate the absorption spectra related to the excited states in PYFR and PYFD1–PYFD7. This analysis provided insights into the probability of charge transfer, configurations driving these transitions and the inherent nature of electronic transitions in these systems. Table 5 shows the calculations for the maximum absorption wavelengths (*λ*_max_), excitation energies (*E*), oscillation strengths (*f*_os_) and contributions from the molecular orbitals for the studied compounds. However, a detailed analysis is shown in Tables S19–S34.†Fig. 6 depicts the UV-Vis absorption spectra of the designed compounds, revealing the absorbance peaks in the gas and solvent mediums.

**Table 5:** Wavelength (*λ*), excitation energy (*E*), oscillator strength (*f*_os_) and nature of molecular orbital (MO) contributions of compounds PYFR and PYFD1–PYFD7

Phase	Compounds	DFT *λ* (nm)	*E* (eV)	*f*_os_	MO contributions
Solvent	PYFR	372.347	3.330	1.124	H → L (86%)
	PYFD1	528.807	2.345	0.142	H → L (53%)
	PYFD2	583.592	2.125	0.059	H → L (89%)
	PYFD3	582.195	2.130	0.043	H → L (92%)
	PYFD4	572.147	2.167	0.050	H → L (90%)
	PYFD5	557.408	2.224	0.063	H → L (88%)
	PYFD6	544.818	2.276	0.090	H → L (77%)
	PYFD7	541.345	2.290	0.104	H → L (67%)
Gaseous	PYFR	371.032	3.342	0.886	H → L (90%)
	PYFD1	553.649	2.239	0.013	H → L (97%)
	PYFD2	685.981	1.807	0.008	H → L (99%)
	PYFD3	697.363	1.778	0.005	H → L (99%)
	PYFD4	671.419	1.847	0.007	H → L (97%)
	PYFD5	629.777	1.969	0.007	H → L (98%)
	PYFD6	591.923	2.095	0.009	H → L (98%)
	PYFD7	589.082	2.105	0.008	H → L (98%)

In chloroform solvent, the PYFD2 compound exhibits the highest absorption wavelength at 583.592 nm, which is attributed to its potent electron-withdrawing nitro groups in the acceptor unit, along with the lowest transition energy value of 2.125 eV. The corresponding oscillation strength of 0.059 can be observed with 89% HOMO to LUMO contribution. The presence of the lowest electron-withdrawing acceptor group in PYFD1 might result in the minimum *λ*_max_, specifically a hypochromic shift with a value of 528.807 nm. This shift is accompanied by the highest excitation energy of 2.345 eV. To enhance the maximum absorption wavelength (*λ*_max_) in the designed compounds, electron-withdrawing acceptor moieties are utilized. The presence of effective electron-withdrawing end-capped acceptors in the compounds leads to a red-shift in the absorption spectrum, causing a change in the absorption maxima (*λ*_max_) towards longer wavelengths. PYFD4 has a slightly longer absorption wavelength at 572.147 *nm* and a higher transition energy of 2.167 eV as compared to PYFD2. This change can be attributed to the addition of electron-withdrawing cyano groups. PYFD5 exhibits a higher absorption wavelength at 557.408 nm as compared to PYFD6. This difference is attributed to the replacement of trifluoromethyl groups with chlorine, potentially resulting in a relatively longer wavelength in the UV region in PYFD5. The lower *λ*_max_ value (541.345 nm) with high transition energy (2.290 eV) observed in PYFD7 as compared to PYFD6 could potentially be attributed to the removal of the chlorine group as it entrapped the charges and the substitution of fluoro groups at the terminal acceptor. This electron-withdrawing effect reduces resonance, resulting in a higher band gap. The descending order of *λ*_max_ values in *nm* is as follows: PYFD2 (583.592) > PYFD3 (582.195) > PYFD4 (572.147) > PYFD5 (557.408) > PYFD6 (544.818) > PYFD7 (541.345) > PYFD1 (528.807) > PYFR (372.347).

The calculated *λ*_max_ values for the designed derivatives consistently exhibited higher values when measured in the gas phase as compared to chloroform. The *λ*_max_ values calculated in the gaseous phase for all the studied compounds fall within the range of 553–697 nm, which is higher than the *λ*_max_ value recorded for PYFR (371.032 nm). Among the reference and designed compounds, PYFD3 displayed the most significant absorption peak at 697.363 nm and the lowest excitation energy of 1.778 eV. This can be ascribed to the introduction of sulphonic acid (–SO_3_H) groups into the acceptor moiety. Their presence leads to a higher absorption peak due to its strong electron-withdrawing nature, enhancing the charge transfer interactions and influencing electronic transitions. The following decreasing order is obtained in nm in the gaseous phase: PYFD3 (697.363) > PYFD2 (685.981) > PYFD4 (671.419) > PYFD5 (629.777) > PYFD6 (591.923) > PYFD7 (589.082) > PYFD1 (553.649) > PYFR (371.032).

Overall, a red-shift is depicted in the designed D–π–A organic molecules as compared to the reference molecule. PYFD2 and PYFD3 stand out as the most suitable candidates due to their prominent *λ*_max_ values and lowest transition energies. Therefore, they are predicted to be favorable NLO materials.

### Nonlinear optical properties

NLO materials are widely used in the optoelectronic devices, telecommunications, optical interconnections, signal and networking manipulation. The strength of the optical response is related to the electrical properties of material, which are influenced by both linear and non-linear properties, *i.e.*, 〈*α*〉, *β*_tot_ and *γ*_tot._^53^ In the case of organic molecules, the establishment of NLO response relies on the asymmetric polarization caused by the push–pull architecture of organic chromophores.^54^ However, the strength of asymmetric polarization is influenced by the chemical nature of various donor and acceptor components, which are connected *via* the π-spacer in the D–π–A framework. This research is fascinating as it depicts linear relationship among the linear and nonlinear responses, hence estimating the optical engagements in the studied chromophores. Table 6 lists the major DFT calculated values of the dipole moment (*μ*_total_), average linear polarizability (〈*α*〉), and first (*β*_tot_) and second hyper-polarizabilities (*γ*_tot_) of the investigated compounds (PYFR and PYFD1–PYFD7), while Tables S35–S38† show their contributing tensors.

**Table 6:** Computed *μ*_total_, 〈*α*〉, *β*_tot_ and *γ*_tot_ values of the considered compounds PYFR and PYFD1–PYFD7^a^

Compounds	*μ*_total_	〈*α*〉 × 10^−22^	*β*_tot_ × 10^−28^	*γ*_tot_ × 10^−33^
PYFR	6.127	1.072	0.165	1.077
PYFD1	10.013	1.750	2.036	2.921
PYFD2	16.062	1.861	4.937	4.631
PYFD3	17.780	1.899	4.128	4.017
PYFD4	16.438	1.872	4.365	4.269
PYFD5	13.105	1.819	3.445	3.529
PYFD6	11.290	1.834	2.431	3.467
PYFD7	10.841	1.752	2.204	3.018

a *μ*_total_ units = Debye (D), while, 〈*α*〉, *β*_tot_ and *γ*_tot_ units = esu.

The electronegativity and polarity of a substance can cause the dipole moments; the larger electronegativity difference marked the higher dipole moments (*μ*).^55^ Moreover, *μ*_total_ is known as a three-dimensional parameter, which established a relationship between the positive and negative centers of charges in a molecule and their influence over the intra-molecular charge transfer (ICT).^56^ The calculated *μ*_total_ of the reference compound (PYFR) is 6.127 *D*, while the derivatives (PYFD1–PYFD7) exhibited larger *μ*_total_ values in the range of 10.013–17.780 D. The increase in *μ*_total_ is significantly attributed to the modification in the D–π–D configuration of PYFR into the D–π–A configuration in the designed compounds, accompanied by the incorporation of potent electron-withdrawing groups. The highest dipole moments are seen in PYFD3 (17.780 D) and PYFD4 (16.438 D) due to the presence of strong electron-withdrawing groups such as –SO_3_H and –CN, respectively. The changes in the dipole moments (beside the *x*, *y* and *z* directions) were also studied. Their contributing tensor values are recorded in Table S35,† which shows that the highest contributing tensor is *μ*_*z*_, *i.e*., 5.677 and 5.028 D obtained for PYFD3 and PYFD4, respectively. In addition, a relative analysis was performed for gaining deeper insight into the polarity of PYFD1–PYFD7. For this purpose, the standard molecule considered is the *para*-nitroaniline chromophore (4.9662 D). The comparison shows that the designed derivatives demonstrated superior polarity as compared to the standard compound, *i.e*., 1.233, 2.016, 3.234, 3.580, 3.310, 2.638, 2.273 and 2.183 times higher *μ*_total_ are obtained for PYFD1–PYFD7 as compared to that of *para*-nitroaniline.

The average linear polarizability (〈*α*〉) predicts the linear optical activity of a molecule, which determines the rate of the intramolecular charge transfer (ICT).^57^ Table S36† shows the detailed results, which include the cartesian coordinates (*x*, *y*, *z*) components, along with the average linear polarizability isotropies for the designed compounds. The conversion factor employed for this purpose is 1 a.u = 1.4819 × 10^−25^ esu. The 〈*α*〉 values are obtained as 1.072, 1.750, 1.861, 1.899, 1.872, 1.819, 1.834 and 1.752 × 10^−22^ esu for the PYFR and PYFD1–PYFD7 compounds, respectively. The impact of end-capped acceptors is predominantly observed in the designed organic chromophores (PYFD1–PYFD7). The highest 〈*α*〉 is obtained for PYFD3 as 1.899 × 10^−22^ esu. The results also showed that the linear polarizability is dominant along the *x*-axis, as indicated by the higher values of *α*_*xx*_ tensors as compared to *α*_*yy*_ and *α*_*zz*_ (Table S36†). This also proved that the maximum intramolecular charge transfer occurs in the x-orientation for the studied molecules. The average polarizability decreases in the following order: PYFD3 > PYFD4 > PYFD2 > PYFD6 > PYFD5 > PYFD7 > PYFD1 > PYFR. This tendency suggests that all developed compounds are more potent than the reference chromophore (PYFR).

Tables S37 and S38† presented the average values alongside with their respective tensor coordinates in the x, *y* and *z* directions. The results showed that the *β*_tot_ values were enhanced with the presence of electron-withdrawing acceptor substituents in the case of PYFD1–PYFD7, hence contributing to a significant NLO response. PYFD2 exhibits the most potent electron-withdrawing groups (–NO_2_) in the 2-(2-methylene-5,6-dinitro-1-oxo-1,2-dihydrocyclopenta[*a*]inden-3(8-*H*)-ylidene)malononitrile acceptor moiety in comparison to other chromophores, leading to the highest *β*_tot_ value (4.937 × 10^−28^ esu). The decreasing order of *β*_tot_ values in esu is as follows: PYFD2 (4.937 × 10^−28^) > PYFD4 (4.365 × 10^−28^) > PYFD3 (4.128 × 10^−28^) > PYFD5 (4.937 × 10^−28^) > PYFD6 (4.937 × 10^−28^) > PYFD7 (4.937 × 10^−28^) > PYFD1 (4.937 × 10^−28^) > PYFR (4.937 × 10^−28^). Moreover, the *β*_tot_ values are mostly influenced by their diagonal *β*_*xxx*_ component (see Table S34†). The maximum average value of the second hyper-polarizability (*γ*_tot_) is observed for PYFD2 (4.631 × 10^−33^ esu). Just like 〈*α*〉, the *γ*_tot_ is also composed of three components along the 3-D plane (*γ*_*x*_, *γ*_*y*_ and *γ*_*z*_). The greatest amplitude in this case is shown by the *γ*_*x*_ tensor (Table S35†). Overall, the following decreasing order in esu is shown as follows: PYFD2 (4.631 × 10^−33^) > PYFD4 (4.269 × 10^−33^) > PYFD3 (4.017 × 10^−33^) > PYFD5 (3.529 × 10^−33^) > PYFD6 (3.467 × 10^−33^) > PYFD7 (3.018 × 10^−33^) > PYFD1 (2.921 × 10^−33^) > PYFR (1.077 × 10^−33^). It can be concluded from the aforementioned discussion that various categories of acceptors have a remarkable impact in producing significant NLO amplitudes.

Frequency-dependent first hyperpolarizability (*β*(*ω*)) coefficients, including the electro-optic Pockel's effect (EOPE) with *β*(−*ω*; *ω*,0) and the second-harmonic generation of first hyperpolarizability (SHG) with *β*(−2*ω*; *ω*,*ω*),^58^ is a time-dependent field. From Table S38,† it can be observed that the first hyperpolarizability (*β*(*ω*)) coefficients are dependent on the wavelengths. The EOPE values are largely enhanced as compared to the static first hyperpolarizability values of the PYFR and PYFD1–PYFD7 chromophores. At 1907.21 nm, the EOPE and SHG values are found in the range of 1.823 × 10^−29^–5.048 × 10^−28^ and 2.388 × 10^−29^–6.904 × 10^−28^ e.s.u., respectively, whereas the response is reduced to be 1.650 × 10^−29^–4.937 × 10^−28^ e.s.u. at static wavelength (0.00 nm). All the designed chromophores have their maximum EOPE and SHG values at 1907.21 nm, indicating the resonant enhancement for EOPE and SHG of the chromophores. Compound PYFD2 shows the best value of EOPE and SHG at 1907.21 nm, which is found to be 5.048 × 10^−28^ and 6.904 × 10^−28^ e.s.u. Similar to the third order nonlinear optical response coefficients (the dc-Kerr effect *γ*(−*ω*; *ω*,0,0)), the electric field induced a second harmonic generation (ESHG) *γ*(−2*ω*; *ω*,*ω*,0)^59^ due to the application of the time-dependent field. The computed results are listed in Table S40,† where one can be seen that all of the designed derivatives show a large dc-Kerr effect *γ*(−*ω*; *ω*,0,0) and electric field-induced second harmonic generation (ESHG) *γ*(−2*ω*; *ω*,*ω*,0) values at the specific frequency. The enormously higher dc-Kerr effect and ESHG values are observed for the PYFD2 derivative, which are found to be 4.883 × 10^−33^ and 7.539 × 10^−33^, respectively, at 1907.21 nm, indicating that both dc-Kerr effect and ESHG values can be remarkably enhanced at higher wavelength for the designed derivatives.

Additionally, a comparative study is made between our designed chromophores with the reported findings of compounds DFPPC and DCPPC.^60^ The findings of the designed chromophores (PYFR and PYFD1–PYFD7) show remarkable results in terms of the linear polarizability and second hyperpolarizability values as compared to the DFPPC and DCPPC compounds. Specifically, the linear polarizability values of the designed chromophores (PYFR and PYFD1–PYFD7) were also found to be 4.10, 6.69, 7.12, 7.28, 7.16, 6.97, 7.02 and 6.71 times greater than that for compound DFPPC (2.6116 × 10^−23^ esu), respectively, and 3.491, 5.69, 6.06, 6.18, 6.10, 5.92, 5.97 and 5.70 times greater than that for compound DCPPC (3.0772 × 10^−23^ esu), respectively. Similarly, the nonlinear second hyperpolarizability values of the designed derivatives (PYFR and PYFD1–PYFD7) were observed to be 33.19, 89.93, 142.78, 123.73, 131.45, 108.60, 106.80 and 92.96 times greater than that for DFPPC (3.2455 × 10^−35^ esu), respectively, and 35.05, 95.04, 150.45, 130.76, 138.99, 114.86, 112.99 and 98.28 times greater than that for DCPPC (3.0708 × 10^−35^ esu), respectively.

By comparing the *β*_*tot*_ values of the designed compound (PYFR and PYFD1–PYFD7) with *para*-nitroaniline, it is observed that the *β*_*tot*_ values of the designed derivatives (PYFD1–PYFD7) were 255, 31.51, 76.31, 63.96, 67.51, 53.28, 37.60 and 34.13 times greater than that of *para*-nitroaniline (*β*_*tot*_ = 6.46 × 10^−30^ esu), respectively. Moreover, the *γ*_tot_ values of the designed derivatives are 147.5, 400.41, 635.25, 550.07, 585.58, 484, 475.4 and 410 times greater than that of the standard compound (*para*-nitroaniline, *γ*_*tot*_ = 7.29 × 10^−36^), respectively.^61^

## Conclusion

The current study consists of a series of compounds, namely PYFD1–PYFD7 and reference compound (PYFR). The strategically placed thiophene molecules, by replacing the benzene spacer unit, as well as various acceptors at one end, were substituted in all structures to create a push–pull architecture to achieve a higher non-linear optical (NLO) response. A quantum chemical investigation was conducted to examine the impact of molecular engineering for NLO characteristics. All of the designed chromophores showed a wider absorption range in the visible region, with a decreased range of energy gap (2.340–2.604 eV). Consequently, they demonstrated efficient charge transfer from the highest occupied molecular orbital (HOMO) to the lowest unoccupied molecular orbital (LUMO) as compared to the reference chromophore. The softness decreases in the following order: PYFD2 > PYFD3 > PYFD4 > PYFD5 > PYFD6 > PYFD7 > PYFD1 > PYFR. So, it was observed that all of the molecules exhibited higher polarizability. Overall, the decreasing order of *γ*_tot_ values in esu is shown as follows: PYFD2 > PYFD4 > PYFD3 > PYFD5 > PYFD6 > PYFD7 > PYFD1 > PYFR. It can be concluded from the aforementioned discussion that all chromophores exhibited a much higher nonlinear optical (NLO) response as compared to the reference compound. This computational study offers valuable insights for experimental researchers to investigate the potential of these appealing nonlinear optical (NLO) materials in the fields of nonlinear optics and electronics.

## Conflicts of interest

There are no conflicts to declare.

## Supplementary Material

RA-014-D4RA00903G-s001
