# Supplementary material for: Influence of benzothiophene acceptor moieties on the non-linear optical properties of pyreno-based chromophores: first-principles DFT framework
Source: RSC Adv. 2024 May 17;14(23):15964–78. doi: 10.1039/d4ra00903g (PMC11099989; doi:10.1039/d4ra00903g)
Supplement: RA-014-D4RA00903G-s001 [file RA-014-D4RA00903G-s001.pdf]

Electronic Supplementary Material (ESI) for RSC Advances.
This journal is © The Royal Society of Chemistry 2024

Influence of Benzothiophene Acceptor Moieties on Non-linear Optical Properties of Pyreno-Based Chromophores: First Principles DFT Framework

Iqra Shafiq[1,2,] Saadia Haq[1,2,] Tayyaba Javed[1,2,] Saifullah Bullo*[3,] Sarfraz Ahmed,[4,] Norah Alhokbany,[5] Tansir Ahamad[5]

[1]Institute of Chemistry, Khwaja Fareed University of Engineering & Information Technology, Rahim Yar Khan, 64200, Pakistan

[2]Centre for Theoretical and Computational Research, Khwaja Fareed University of Engineering & Information Technology, Rahim Yar Khan, 64200, Pakistan

[3]Department of Human and Rehabilitation Sciences, Begum Nusrat Bhutto Women University, Sukkur Sindh 65170 Pakistan

[4]Wellman Center for Photomedicine, Harvard Medical School, Massachusetts General Hospital, Boston, MA 02114, United States

[5]Department of Chemistry, College of Science, King Saud University, Saudi Arabia

*Corresponding authors E-mail address:

Dr. Saifullah Bullo (saifullah.bullo@bnbwu.edu.pk)

Table S1: Cartesian coordinates of **PYFR** compound.

Atom	X-axis	Y-axis	Z-axis
C	-6.173517	2.889816	-0.316649
C	-4.929739	2.280545	-0.220052
C	-4.819368	0.893489	-0.090428
C	-6.007975	0.099053	-0.090051
C	-7.27005	0.744911	-0.178333
C	-7.331209	2.137578	-0.286519
C	-5.960092	-1.329768	-0.017058
C	-4.721467	-2.022753	0.039196
C	-3.534522	-1.222643	0.04566
C	-4.699175	-3.412646	0.095802
H	-3.738856	-3.917053	0.139678
C	-5.885707	-4.133915	0.101671
C	-7.103577	-3.478359	0.047711
C	-7.164031	-2.082171	-0.015163
C	-8.415657	-1.392339	-0.08362
C	-8.464344	-0.044194	-0.164256
H	-9.417799	0.47412	-0.224557
H	-9.32833	-1.982522	-0.076076
H	-6.23047	3.969122	-0.416255
H	-4.040922	2.899161	-0.254461

H	-8.30434	2.616283	-0.35587
H	-5.856847	-5.218015	0.149021
H	-8.032559	-4.042415	0.050715
C	-3.592949	0.158576	0.019129
C	-1.501261	-0.573351	0.113821
C	-0.037131	-0.59276	0.137359
C	0.617194	-1.667095	-0.472532
C	0.741853	0.387947	0.757192
C	1.997117	-1.74233	-0.488249
H	0.022335	-2.436987	-0.952827
C	2.122171	0.305871	0.741595
H	0.273401	1.216032	1.277662
C	2.781797	-0.751215	0.10965
H	2.476968	-2.569568	-1.003317
H	2.700512	1.067928	1.255616
N	-2.273256	0.572374	0.075596
N	-2.242229	-1.659272	0.094479
C	4.254628	-0.809352	0.076307
C	5.005643	0.367387	-0.04017
C	4.926851	-2.036025	0.16341
H	4.500654	1.325616	-0.136668
C	6.310547	-2.105655	0.141147
H	4.349488	-2.949191	0.277289
C	7.042194	-0.930256	0.026983
H	6.810791	-3.066974	0.221353
C	6.384062	0.304232	-0.065614
C	7.388776	1.41432	-0.195585
H	7.300666	2.146164	0.617604
H	7.258865	1.975716	-1.129837
C	8.481112	-0.695469	-0.024906
C	8.701699	0.683571	-0.156568
C	9.99056	1.181424	-0.230116
C	11.060188	0.292933	-0.170078
H	10.167311	2.248897	-0.33257
C	9.549606	-1.582021	0.034906
C	10.84044	-1.07607	-0.038268
H	12.076867	0.669965	-0.225862
H	9.37995	-2.650631	0.136906
H	11.688249	-1.752819	0.007149
C	-1.814077	1.924446	0.083261
C	-1.928149	2.670836	1.248434
C	-1.273269	2.472511	-1.069217
C	-1.495621	3.987746	1.256737

H	-2.358874	2.212706	2.134354
C	-0.837499	3.790289	-1.052024
H	-1.197799	1.864704	-1.96574
C	-0.950173	4.546771	0.106851
H	-1.582666	4.578972	2.162269
H	-0.412017	4.228269	-1.949002
H	-0.611446	5.577635	0.114733

Table S2: Cartesian coordinates of **PYFD1** compound.

Atom	X-axis	Y-axis	Z-axis
C	-12.845301	-1.289396	1.369676
C	-11.494762	-1.117557	1.096479
C	-11.014975	0.08848	0.579886
C	-11.933149	1.164869	0.364347
C	-13.31068	0.964717	0.646193
C	-13.746518	-0.268214	1.14083
C	-11.500212	2.442126	-0.114652
C	-10.131917	2.696757	-0.392125
C	-9.220376	1.612451	-0.189553
C	-9.728869	3.949847	-0.841857
H	-8.676225	4.118215	-1.046303
C	-10.661986	4.961376	-1.026455
C	-12.002004	4.734082	-0.765323
C	-12.442194	3.487192	-0.309195
C	-13.822829	3.239847	-0.028116
C	-14.235054	2.036259	0.427909
H	-15.284287	1.854706	0.645974
H	-14.532846	4.047358	-0.186365
H	-13.190271	-2.238321	1.76812
H	-10.812654	-1.934872	1.296488
H	-14.803733	-0.405527	1.351296
H	-10.337633	5.935789	-1.378471
H	-12.732826	5.525466	-0.909598
C	-9.648428	0.373315	0.250117
C	-7.45547	0.413899	-0.094346
C	-6.045126	0.025303	-0.174996
C	-5.093285	1.047921	-0.095179
C	-5.588607	-1.280791	-0.375569
C	-3.744176	0.777757	-0.206295
H	-5.436756	2.067144	0.044025
C	-4.234709	-1.545478	-0.486556
H	-6.287138	-2.105746	-0.458888
C	-3.282282	-0.527459	-0.405466

H	-3.03393	1.598392	-0.161945
H	-3.911984	-2.57324	-0.624303
N	-8.500168	-0.400592	0.301273
N	-7.873135	1.624999	-0.392003
C	-1.843139	-0.818096	-0.540475
C	-1.400178	-1.827931	-1.404112
C	-0.894806	-0.085625	0.186827
H	-2.117304	-2.390537	-1.996871
C	0.462385	-0.337064	0.068154
H	-1.233937	0.68148	0.876956
C	0.886681	-1.339804	-0.794637
H	1.177508	0.240133	0.647738
C	-0.049267	-2.082607	-1.527385
C	0.653806	-3.091311	-2.391071
H	0.438871	-2.940805	-3.456877
H	0.347471	-4.119156	-2.157771
C	2.223159	-1.81965	-1.129529
C	2.100665	-2.850837	-2.072619
C	3.231617	-3.477607	-2.564876
C	4.479697	-3.073088	-2.114817
H	3.150078	-4.282672	-3.28962
C	3.469214	-1.409703	-0.682547
C	4.615547	-2.038859	-1.176873
H	3.555336	-0.590207	0.02692
C	-8.415926	-1.760478	0.728964
C	-8.811542	-2.77337	-0.134502
C	-7.939393	-2.046098	1.998863
C	-8.727188	-4.092335	0.284133
H	-9.182658	-2.517414	-1.122837
C	-7.852459	-3.368999	2.408876
H	-7.636947	-1.232249	2.65092
C	-8.245974	-4.38992	1.553815
H	-9.034443	-4.890768	-0.383198
H	-7.477959	-3.602423	3.400075
H	-8.178501	-5.423396	1.877683
C	8.782113	-0.206132	1.502049
H	8.476822	-0.369251	2.538271
C	9.990694	0.392365	1.38339
C	10.744669	0.930715	0.247952
C	10.873169	0.442092	2.593155
C	12.13916	0.977329	0.671879
C	10.230481	1.511779	-0.878862
C	12.180476	0.755457	2.017832

O	13.39535	0.858332	2.552677
C	13.497766	1.249759	0.285922
H	5.367985	-3.580639	-2.478129
C	14.219795	1.152107	1.49304
C	15.585526	1.319598	1.593882
C	16.258537	1.589019	0.415022
H	16.09021	1.236453	2.549201
C	14.210554	1.509453	-0.889879
C	15.578787	1.678109	-0.806195
H	17.334075	1.728232	0.437627
H	13.705306	1.584128	-1.844505
H	16.143243	1.883987	-1.709418
C	8.837827	1.715313	-1.082913
N	7.72124	1.942346	-1.270102
C	11.058103	2.087564	-1.879956
N	11.697387	2.56121	-2.717089
O	10.568615	0.224883	3.740532
C	8.239367	-1.248758	-0.743982
C	5.950197	-1.639555	-0.741008
C	7.922331	-0.736482	0.495197
S	6.217679	-0.92255	0.812882
C	7.133686	-1.757552	-1.435253
H	7.190049	-2.183093	-2.430232
H	9.253878	-1.278422	-1.125584

Table S3: Cartesian coordinates of **PYFD2** compound.

Atom	X-axis	Y-axis	Z-axis
C	-14.446241	-1.172886	1.430573
C	-13.095283	-1.018317	1.149324
C	-12.608502	0.169914	0.598424
C	-13.520169	1.245138	0.352203
C	-14.898249	1.062562	0.64336
C	-15.340898	-0.152341	1.175257
C	-13.080589	2.503958	-0.16803
C	-11.711674	2.741003	-0.458116
C	-10.806317	1.657657	-0.224324
C	-11.30246	3.976756	-0.94852
H	-10.24953	4.131864	-1.161732
C	-12.229775	4.988054	-1.161775
C	-13.570248	4.77752	-0.889294
C	-14.016555	3.548396	-0.392594
C	-15.397828	3.318891	-0.099649
C	-15.816334	2.132796	0.39484

H	-16.866045	1.964931	0.621392
H	-16.103159	4.125639	-0.281012
H	-14.796705	-2.107872	1.856238
H	-12.418717	-1.835165	1.369554
H	-16.398433	-0.276138	1.392409
H	-11.900515	5.948742	-1.54548
H	-14.296697	5.568508	-1.056039
C	-11.24083	0.436747	0.258175
C	-9.048737	0.449883	-0.096502
C	-7.64037	0.051874	-0.172261
C	-6.687236	1.07714	-0.191585
C	-7.183986	-1.265738	-0.274995
C	-5.339948	0.799293	-0.30418
H	-7.028856	2.104428	-0.129847
C	-5.831471	-1.538264	-0.386492
H	-7.879316	-2.096692	-0.281194
C	-4.87918	-0.5175	-0.404976
H	-4.631169	1.621595	-0.339868
H	-5.509931	-2.573927	-0.445789
N	-10.097762	-0.342436	0.331847
N	-9.46049	1.653124	-0.433585
C	-3.442463	-0.818948	-0.541618
C	-3.01407	-1.892029	-1.3328
C	-2.482746	-0.034893	0.113646
H	-3.741168	-2.496409	-1.86943
C	-1.127772	-0.297604	-0.005288
H	-2.811227	0.783479	0.747638
C	-0.717937	-1.364335	-0.795114
H	-0.40344	0.319937	0.518542
C	-1.665315	-2.158112	-1.456342
C	-0.976733	-3.232526	-2.24925
H	-1.203812	-3.163118	-3.32082
H	-1.283267	-4.238425	-1.934784
C	0.612405	-1.874915	-1.108458
C	0.474507	-2.975876	-1.967567
C	1.595609	-3.647738	-2.421705
C	2.851113	-3.219537	-2.017176
H	1.500438	-4.506073	-3.080405
C	1.865252	-1.442477	-0.706368
C	3.002257	-2.118196	-1.161432
H	1.96385	-0.572652	-0.061423
C	-10.029008	-1.690083	0.79909
C	-10.41821	-2.72313	-0.042793

C	-9.578622	-1.9431	2.085455
C	-10.352587	-4.030623	0.413583
H	-10.769634	-2.491144	-1.044081
C	-9.510818	-3.254609	2.533566
H	-9.281653	-1.113289	2.719672
C	-9.897221	-4.295924	1.699871
H	-10.654804	-4.845152	-0.236309
H	-9.15703	-3.46335	3.537801
H	-9.8444	-5.3203	2.05402
C	7.160279	-0.115773	1.367631
H	6.756633	-0.07733	2.382433
C	8.441917	0.338622	1.297282
C	9.361556	0.623246	0.199771
C	9.185017	0.478286	2.584355
C	10.705719	0.623936	0.78133
C	9.046235	1.056647	-1.058675
C	10.579459	0.59444	2.135739
O	11.732151	0.674282	2.801175
C	12.119341	0.729883	0.54053
H	3.731576	-3.760539	-2.349425
C	12.684101	0.751343	1.831769
C	14.038439	0.815732	2.086864
C	14.860262	0.861494	0.9845
H	14.443319	0.799546	3.09105
C	12.984061	0.778047	-0.556618
C	14.335512	0.850966	-0.315618
H	12.625056	0.791298	-1.578615
C	7.718866	1.367801	-1.458893
N	6.656731	1.666414	-1.79894
C	10.046712	1.356702	-2.021069
N	10.843441	1.597302	-2.821553
O	8.749866	0.455473	3.708081
C	6.634849	-1.348082	-0.793009
C	4.340503	-1.701032	-0.759737
C	6.32105	-0.713897	0.395577
S	4.60863	-0.845462	0.716488
C	5.528613	-1.904304	-1.435023
H	5.582928	-2.423355	-2.384418
H	7.649124	-1.427684	-1.168106
N	15.202483	1.055999	-1.492133
N	16.309441	0.77798	1.234398
O	16.054542	1.910036	-1.400413
O	16.73538	1.362959	2.203413

O	16.95672	0.090678	0.477288
O	14.963603	0.392286	-2.473944

Table S4: Cartesian coordinates of **PYFD3** compound.

Atom	Atom2	Y-axis	Z-axis
C	-15.530832	-1.130152	1.428057
C	-14.180454	-0.977484	1.143095
C	-13.691458	0.214705	0.602923
C	-14.599934	1.296137	0.372708
C	-15.977558	1.115387	0.667162
C	-16.422713	-0.103675	1.187256
C	-14.157387	2.55933	-0.134208
C	-12.788605	2.794717	-0.42644
C	-11.886589	1.70506	-0.209388
C	-12.376488	4.034935	-0.90292
H	-11.323739	4.188805	-1.11793
C	-13.300728	5.052257	-1.100295
C	-14.641024	4.843373	-0.825752
C	-15.090211	3.609953	-0.342556
C	-16.471275	3.382165	-0.047324
C	-16.892513	2.191882	0.434634
H	-17.942003	2.025378	0.663205
H	-17.174151	4.193718	-0.21636
H	-15.883047	-2.068379	1.845053
H	-13.506089	-1.799063	1.35202
H	-17.479832	-0.225925	1.40727
H	-12.96915	6.016422	-1.473135
H	-15.365045	5.639085	-0.980101
C	-12.324245	0.480094	0.259849
C	-10.133517	0.488942	-0.102386
C	-8.727387	0.085115	-0.186022
C	-7.767659	1.10426	-0.18137
C	-8.279888	-1.232944	-0.317115
C	-6.421859	0.819866	-0.29745
H	-8.102826	2.132167	-0.09727
C	-6.92922	-1.512037	-0.431994
H	-8.981283	-2.058484	-0.342882
C	-5.9699	-0.497608	-0.425986
H	-5.707251	1.637788	-0.312996
H	-6.614717	-2.548351	-0.513033
N	-11.184192	-0.304626	0.319506
N	-10.541316	1.697837	-0.423291
C	-4.535348	-0.808422	-0.564648

C	-4.115069	-1.871827	-1.373116
C	-3.569792	-0.046855	0.108171
H	-4.846041	-2.458633	-1.923753
C	-2.217298	-0.323781	-0.008323
H	-3.891686	0.764016	0.755105
C	-1.815728	-1.382349	-0.813337
H	-1.488656	0.275822	0.530173
C	-2.769124	-2.152189	-1.493846
C	-2.090051	-3.222765	-2.299813
H	-2.306309	-3.13033	-3.371936
H	-2.414705	-4.22939	-2.006694
C	-0.490363	-1.907496	-1.124401
C	-0.637821	-2.993155	-2.001331
C	0.47633	-3.676813	-2.454652
C	1.734559	-3.275396	-2.03194
H	0.373414	-4.523894	-3.126634
C	0.765602	-1.501124	-0.704851
C	1.895683	-2.18925	-1.158651
H	0.872143	-0.642782	-0.045891
C	-11.11752	-1.656806	0.773916
C	-11.515846	-2.681312	-0.074202
C	-10.659013	-1.922652	2.054738
C	-11.45151	-3.992888	0.370556
H	-11.873259	-2.43981	-1.071135
C	-10.59254	-3.23819	2.491176
H	-10.354853	-1.099434	2.694129
C	-10.98821	-4.270964	1.651281
H	-11.760861	-4.800692	-0.284382
H	-10.232459	-3.456704	3.491082
H	-10.936437	-5.298587	1.996008
C	6.071089	-0.30484	1.435938
H	5.665769	-0.279927	2.450447
C	7.360602	0.128674	1.375231
C	8.283116	0.418042	0.282035
C	8.108976	0.222872	2.665075
C	9.625026	0.39253	0.863927
C	7.977745	0.863509	-0.976222
C	9.505154	0.328497	2.217392
O	10.662923	0.389853	2.878613
C	11.036021	0.514939	0.622191
H	2.608752	-3.8269	-2.363286
C	11.60926	0.495475	1.903497
C	12.965135	0.578381	2.139743

C	13.79226	0.691076	1.0376
H	13.35129	0.578915	3.150778
C	11.890996	0.602556	-0.474626
C	13.252037	0.692449	-0.26883
H	11.494012	0.58896	-1.478888
C	6.658461	1.207567	-1.375481
N	5.603941	1.533756	-1.714068
C	8.984259	1.127774	-1.943552
N	9.78969	1.32864	-2.746771
O	7.673257	0.179303	3.787837
C	5.538407	-1.496193	-0.743746
C	3.237252	-1.8024	-0.738
C	5.225721	-0.869614	0.44904
S	3.507706	-0.96996	0.751402
C	4.427819	-2.021773	-1.403663
H	4.481841	-2.530758	-2.358481
H	6.554812	-1.592994	-1.108929
S	15.558286	0.784132	1.342542
S	14.273033	0.849811	-1.743371
O	13.30226	0.239037	-2.868354
O	14.474506	2.243221	-2.048704
O	15.362027	-0.080647	-1.630719
O	16.114581	-0.543538	1.421199
O	15.572891	1.406588	2.821994
O	16.099177	1.787946	0.468774
H	15.923976	0.758222	3.451422
H	12.86517	0.947272	-3.367426

Table S5: Cartesian coordinates of **PYFD4** compound.

Atom	X-axis	Y-axis	Z-axis
C	-13.808272	-1.200321	1.417543
C	-12.455735	-1.043596	1.145172
C	-11.969421	0.139981	0.583807
C	-12.883525	1.208037	0.316538
C	-14.263128	1.023348	0.599063
C	-14.704966	-0.186691	1.142658
C	-12.445142	2.461754	-0.216925
C	-11.074976	2.700751	-0.49948
C	-10.166904	1.624677	-0.243761
C	-10.6672	3.93125	-1.00413
H	-9.613305	4.087772	-1.211514
C	-11.597089	4.935443	-1.238741
C	-12.93873	4.722987	-0.973501

C	-13.383695	3.498909	-0.463223
C	-14.76627	3.26738	-0.178012
C	-15.183661	2.086236	0.329105
H	-16.234447	1.916822	0.549434
H	-15.473577	4.068465	-0.376014
H	-14.15836	-2.131498	1.851787
H	-11.777619	-1.85496	1.380565
H	-15.763724	-0.312277	1.352733
H	-11.268963	5.892009	-1.633545
H	-13.667211	5.508379	-1.156989
C	-10.600124	0.408368	0.251341
C	-8.405507	0.425607	-0.087614
C	-6.995079	0.032293	-0.150168
C	-6.04663	1.061469	-0.187363
C	-6.531844	-1.284835	-0.225267
C	-4.697849	0.787917	-0.29121
H	-6.393201	2.088143	-0.147479
C	-5.177603	-1.553018	-0.327447
H	-7.222674	-2.119427	-0.216957
C	-4.230182	-0.528277	-0.364406
H	-3.993307	1.612962	-0.342651
H	-4.851139	-2.588158	-0.365204
N	-9.454555	-0.365049	0.343828
N	-8.819669	1.622774	-0.4432
C	-2.791473	-0.823488	-0.493405
C	-2.355007	-1.911682	-1.259092
C	-1.837528	-0.015884	0.141653
H	-3.077637	-2.534987	-1.779832
C	-0.480489	-0.268191	0.024855
H	-2.172162	0.814085	0.757014
C	-0.062621	-1.348889	-0.741419
H	0.239377	0.368555	0.531564
C	-1.004097	-2.168365	-1.379355
C	-0.307234	-3.255581	-2.147194
H	-0.540882	-3.218528	-3.218897
H	-0.600361	-4.255803	-1.803148
C	1.27194	-1.851154	-1.050079
C	1.142428	-2.97486	-1.880487
C	2.269073	-3.64366	-2.325434
C	3.521766	-3.188699	-1.941926
H	2.180555	-4.519524	-2.961578
C	2.521908	-1.391994	-0.669393
C	3.664778	-2.06283	-1.11719

H	2.613304	-0.504611	-0.047746
C	-9.385932	-1.708135	0.823952
C	-9.764285	-2.749175	-0.012935
C	-8.947227	-1.948961	2.116748
C	-9.699203	-4.052684	0.454676
H	-10.106867	-2.526198	-1.019301
C	-8.879911	-3.256582	2.576162
H	-8.658592	-1.113032	2.746748
C	-9.25532	-4.305868	1.747431
H	-9.992957	-4.873488	-0.19116
H	-8.535126	-3.456066	3.585404
H	-9.202847	-5.32707	2.110719
C	7.808204	0.096573	1.302705
H	7.3936	0.190153	2.309549
C	9.092232	0.543286	1.22636
C	10.034417	0.756991	0.131605
C	9.814776	0.750187	2.51651
C	11.368017	0.77416	0.736137
C	9.744027	1.122542	-1.1545
C	11.217547	0.825226	2.08729
O	12.358991	0.93064	2.768381
C	12.787409	0.851053	0.514434
H	4.406656	-3.726138	-2.268057
C	13.33102	0.936935	1.813549
C	14.678192	1.009554	2.091182
C	15.536874	0.98434	1.002996
H	15.046039	1.078642	3.10716
C	13.6683	0.815209	-0.567139
C	15.030126	0.883706	-0.317679
H	13.313819	0.74341	-1.58775
C	8.426588	1.427511	-1.590514
N	7.373678	1.720353	-1.962911
C	10.760337	1.3561	-2.118858
N	11.568456	1.54155	-2.922576
O	9.359974	0.796577	3.631898
C	7.289259	-1.2304	-0.805895
C	4.999982	-1.613061	-0.74132
C	6.974989	-0.551724	0.357964
S	5.266168	-0.692655	0.69536
C	6.186524	-1.826918	-1.416608
H	6.242473	-2.38283	-2.34469
H	8.301329	-1.310481	-1.186639
C	15.939685	0.849882	-1.41683

N	16.678316	0.821623	-2.301998
C	16.943751	1.055991	1.222653
N	18.081724	1.114412	1.40042

Table S6: Cartesian coordinates of **PYFD5** compound.

Atom	X-axis	Y-axis	Z-axis
C	-15.081973	-1.183057	1.446404
C	-13.731214	-1.025914	1.165586
C	-13.24702	0.16201	0.611735
C	-14.161259	1.234214	0.361789
C	-15.539058	1.049046	0.652602
C	-15.97901	-0.165447	1.187691
C	-13.724588	2.492542	-0.162056
C	-12.35614	2.732079	-0.45215
C	-11.448222	1.65174	-0.214581
C	-11.949705	3.967209	-0.946433
H	-10.897057	4.124179	-1.159662
C	-12.879418	4.975483	-1.163503
C	-14.219508	4.762507	-0.89101
C	-14.663038	3.533892	-0.390548
C	-16.043891	3.301817	-0.097691
C	-16.459677	2.116209	0.400239
H	-17.509101	1.946357	0.626644
H	-16.751179	4.106169	-0.282045
H	-15.430396	-2.117729	1.874435
H	-13.052738	-1.840466	1.388423
H	-17.036377	-0.29129	1.404494
H	-12.552376	5.935705	-1.550255
H	-14.947842	5.551117	-1.060782
C	-11.879803	0.431232	0.271505
C	-9.687712	0.448527	-0.083553
C	-8.278427	0.053855	-0.160403
C	-7.32845	1.08186	-0.189798
C	-7.818179	-1.262948	-0.256135
C	-5.980819	0.807501	-0.306108
H	-7.67297	2.108468	-0.133505
C	-6.465206	-1.531965	-0.371259
H	-8.510669	-2.096253	-0.254235
C	-5.516187	-0.508424	-0.400527
H	-5.274937	1.631857	-0.350262
H	-6.14076	-2.567019	-0.425098
N	-10.734778	-0.344931	0.347537
N	-10.102492	1.649813	-0.424011

C	-4.079049	-0.805633	-0.542069
C	-3.650447	-1.882327	-1.328202
C	-3.119076	-0.01349	0.103059
H	-4.377904	-2.493152	-1.856989
C	-1.763671	-0.271509	-0.021343
H	-3.447492	0.807701	0.733422
C	-1.353625	-1.341789	-0.80624
H	-1.039065	0.352303	0.494629
C	-2.301299	-2.143953	-1.456916
C	-1.612595	-3.221547	-2.24537
H	-1.845286	-3.160854	-3.316264
H	-1.914079	-4.226109	-1.921864
C	-0.022854	-1.849493	-1.122672
C	-0.160946	-2.957503	-1.972453
C	0.960547	-3.629247	-2.42575
C	2.216288	-3.194171	-2.029275
H	0.865576	-4.493223	-3.077098
C	1.23039	-1.409457	-0.729819
C	2.367894	-2.085114	-1.183626
H	1.328551	-0.534417	-0.091865
C	-10.663312	-1.691536	0.817344
C	-11.049669	-2.726785	-0.023109
C	-10.213563	-1.941392	2.104585
C	-10.981761	-4.033372	0.435491
H	-11.400715	-2.497137	-1.025059
C	-10.143475	-3.252008	2.554935
H	-9.918806	-1.109885	2.737608
C	-10.527011	-4.29552	1.722649
H	-11.281731	-4.849639	-0.213252
H	-9.790133	-3.458342	3.559826
H	-10.472415	-5.319169	2.07864
C	6.528046	-0.057047	1.32501
H	6.112549	0.003742	2.334004
C	7.816163	0.375654	1.266365
C	8.764998	0.611176	0.180565
C	8.541972	0.52389	2.5646
C	10.09602	0.583134	0.786551
C	8.482161	1.021476	-1.093739
C	9.944813	0.586166	2.139481
O	11.087778	0.64235	2.823519
C	11.517658	0.642968	0.570239
H	3.096896	-3.73559	-2.360443
C	12.059165	0.66561	1.864557

C	13.409909	0.695934	2.132576
C	14.277522	0.700367	1.055317
H	13.769414	0.712677	3.153035
C	12.40869	0.635599	-0.503556
C	13.770636	0.661672	-0.266573
H	12.044899	0.613746	-1.521359
C	7.170527	1.361883	-1.521146
N	6.12321	1.68359	-1.885515
C	9.50327	1.265586	-2.050751
N	10.313925	1.459738	-2.849923
O	8.083683	0.538972	3.67975
C	6.00248	-1.314099	-0.824062
C	3.706698	-1.661292	-0.789549
C	5.689449	-0.661862	0.353935
S	3.976044	-0.782857	0.673441
C	4.894626	-1.875732	-1.460403
H	4.94956	-2.408285	-2.402265
H	7.016699	-1.402138	-1.197286
C	14.660073	0.708022	-1.485032
C	15.750251	0.675092	1.38797
F	15.139167	1.937098	-1.694682
F	15.702717	-0.118525	-1.387686
F	13.997105	0.361013	-2.589494
F	16.463286	1.509902	0.634156
F	15.962696	1.026685	2.65809
F	16.263485	-0.548349	1.237882

Table S7: Cartesian coordinates of **PYFD6** compound.

Atom	X-axis	Y-axis	Z-axis
C	-14.033854	-1.167177	1.43015
C	-12.683001	-1.014905	1.147166
C	-12.195582	0.170934	0.591599
C	-13.10652	1.245987	0.341987
C	-14.484517	1.065735	0.634996
C	-14.92779	-0.146751	1.171885
C	-12.666291	2.502383	-0.183555
C	-11.297383	2.737183	-0.475556
C	-10.392705	1.654095	-0.238003
C	-10.887556	3.970641	-0.971242
H	-9.834655	4.124013	-1.185856
C	-11.814215	4.981814	-1.187918
C	-13.154653	4.773454	-0.913584
C	-13.601559	3.54669	-0.411591

C	-14.98278	3.319511	-0.116576
C	-15.401874	2.13578	0.383047
H	-16.451537	1.969736	0.61117
H	-15.687586	4.12612	-0.300589
H	-14.384804	-2.100278	1.859545
H	-12.00702	-1.831634	1.369699
H	-15.985245	-0.268753	1.390442
H	-11.484472	5.940674	-1.575754
H	-13.880602	5.564343	-1.082963
C	-10.827915	0.435449	0.249575
C	-8.635964	0.4454	-0.106401
C	-7.227895	0.046061	-0.180859
C	-6.274151	1.070654	-0.206351
C	-6.772151	-1.272335	-0.276168
C	-4.926999	0.791442	-0.317128
H	-6.615194	2.09848	-0.150682
C	-5.419725	-1.546218	-0.385725
H	-7.467764	-2.103033	-0.277745
C	-4.466791	-0.526168	-0.409783
H	-4.217786	1.613146	-0.357607
H	-5.098707	-2.582383	-0.438872
N	-9.685414	-0.3443	0.32578
N	-9.047016	1.647621	-0.448072
C	-3.030109	-0.829248	-0.542946
C	-2.60113	-1.907742	-1.326475
C	-2.070929	-0.041745	0.10888
H	-3.327831	-2.515016	-1.860408
C	-0.715936	-0.30647	-0.00576
H	-2.399893	0.780926	0.737046
C	-0.305478	-1.378771	-0.787688
H	0.007967	0.313687	0.51557
C	-1.252405	-2.175935	-1.445638
C	-0.56322	-3.256811	-2.229295
H	-0.787676	-3.194288	-3.301853
H	-0.872644	-4.259853	-1.908541
C	1.025011	-1.893418	-1.093966
C	0.887773	-3.000244	-1.945147
C	2.009583	-3.677367	-2.389679
C	3.264388	-3.248848	-1.982706
H	1.915559	-4.540638	-3.042167
C	2.277415	-1.459802	-0.690895
C	3.414995	-2.141085	-1.13542
H	2.375119	-0.585441	-0.051929

C	-9.618112	-1.690627	0.796994
C	-10.008013	-2.725536	-0.04225
C	-9.168616	-1.940562	2.084292
C	-9.943903	-4.031867	0.417636
H	-10.358729	-2.495827	-1.044306
C	-9.102333	-3.250932	2.535929
H	-8.871085	-1.109324	2.716371
C	-9.489362	-4.294113	1.704846
H	-10.246632	-4.847865	-0.230166
H	-8.749187	-3.457338	3.540875
H	-9.437642	-5.317567	2.061822
C	7.565096	-0.121273	1.404928
H	7.17336	-0.100552	2.424888
C	8.82939	0.365508	1.31848
C	9.731895	0.667093	0.207687
C	9.589721	0.531877	2.596128
C	11.07994	0.688996	0.77086
C	9.387294	1.094207	-1.045788
C	10.970432	0.669265	2.129056
O	12.129701	0.780102	2.777371
C	12.488349	0.824407	0.506433
H	4.145244	-3.794359	-2.306638
C	13.070887	0.86569	1.787932
C	14.424382	0.970207	2.018884
C	15.24197	1.0247	0.904913
H	14.830889	1.002549	3.021951
C	13.336796	0.868606	-0.600652
C	14.698101	0.970369	-0.391682
H	12.956409	0.83415	-1.613923
C	8.047518	1.377703	-1.425962
N	6.976049	1.656305	-1.753939
C	10.362969	1.418322	-2.02609
N	11.132144	1.679646	-2.84696
O	9.162488	0.509701	3.724137
C	7.048758	-1.373824	-0.747113
C	4.752989	-1.722985	-0.730848
C	6.727989	-0.733948	0.433478
S	5.014712	-0.859221	0.744081
C	5.943603	-1.931022	-1.395848
H	6.003578	-2.454702	-2.342538
H	8.065702	-1.454746	-1.115065
C1	15.738066	1.026635	-1.781502
C1	16.952207	1.15368	1.145641

Table S8: Cartesian coordinates of **PYFD7** compound.

Atom	X-axis	Y-axis	Z-axis
C	-13.419585	-1.238654	1.364408
C	-12.069247	-1.071091	1.087707
C	-11.585799	0.136145	0.577454
C	-12.499542	1.218386	0.373076
C	-13.877083	1.022382	0.657748
C	-14.316917	-0.211965	1.145239
C	-12.061932	2.497354	-0.097057
C	-10.693112	2.748159	-0.375705
C	-9.786086	1.657874	-0.18487
C	-10.285651	4.003093	-0.816242
H	-9.23275	4.168519	-1.021825
C	-11.214776	5.020108	-0.990685
C	-12.55519	4.79656	-0.728406
C	-12.999769	3.548054	-0.281043
C	-14.380792	3.304656	0.00161
C	-14.797234	2.099617	0.449906
H	-15.846684	1.921235	0.66953
H	-15.087587	4.11649	-0.148765
H	-13.767504	-2.188598	1.757822
H	-11.390172	-1.892722	1.280127
H	-15.374052	-0.345932	1.358235
H	-10.886932	5.995887	-1.335611
H	-13.282877	5.592221	-0.864779
C	-10.219183	0.416973	0.244868
C	-8.02739	0.449043	-0.106758
C	-6.61983	0.052234	-0.19491
C	-5.660343	1.065975	-0.097446
C	-6.17353	-1.253786	-0.417601
C	-4.313071	0.787047	-0.210879
H	-5.996322	2.085335	0.058577
C	-4.821657	-1.527248	-0.53064
H	-6.878715	-2.071425	-0.516045
C	-3.861227	-0.518524	-0.430057
H	-3.596328	1.601057	-0.151235
H	-4.507012	-2.555147	-0.684805
N	-9.075081	-0.363485	0.284906
N	-8.439245	1.665205	-0.391421
C	-2.424188	-0.820684	-0.562593
C	-1.987032	-1.825295	-1.435255
C	-1.472199	-0.107477	0.178953

H	-2.706463	-2.372915	-2.039102
C	-0.117263	-0.3737	0.066545
H	-1.806823	0.655398	0.875965
C	0.301079	-1.372347	-0.803919
H	0.600499	0.188283	0.657705
C	-0.638536	-2.095226	-1.551643
C	0.057983	-3.104712	-2.419644
H	-0.144823	-2.940013	-3.485729
H	-0.264903	-4.130486	-2.200065
C	1.633687	-1.86724	-1.132292
C	1.505067	-2.888173	-2.085792
C	2.631171	-3.527365	-2.573054
C	3.880746	-3.1449	-2.108394
H	2.544568	-4.324932	-3.305433
C	2.881379	-1.479087	-0.6709
C	4.023025	-2.120741	-1.16034
H	2.972619	-0.667177	0.046602
C	-8.996844	-1.729354	0.694364
C	-9.405635	-2.727942	-0.179632
C	-8.51121	-2.03537	1.955977
C	-9.325398	-4.053058	0.220005
H	-9.783384	-2.456337	-1.161276
C	-8.428309	-3.364397	2.346829
H	-8.197991	-1.232533	2.616553
C	-8.834929	-4.371083	1.481145
H	-9.642939	-4.840301	-0.455771
H	-8.046434	-3.613692	3.331324
H	-8.770571	-5.409342	1.789958
C	8.1906	-0.354954	1.559588
H	7.871004	-0.507502	2.593063
C	9.413255	0.219089	1.451736
C	10.184903	0.742808	0.322846
C	10.284929	0.250972	2.669167
C	11.576377	0.772931	0.761483
C	9.693989	1.324026	-0.81389
C	11.60257	0.546577	2.106161
O	12.816441	0.632054	2.651643
C	12.939693	1.033204	0.390883
H	4.764455	-3.662644	-2.468265
C	13.648275	0.919337	1.603324
C	15.016192	1.07106	1.718498
C	15.688011	1.337707	0.548872
H	15.540572	0.98239	2.661731

C	13.659122	1.291875	-0.780672
C	15.017718	1.443157	-0.677325
H	13.189388	1.390388	-1.751158
C	8.308014	1.550231	-1.037475
N	7.19812	1.795385	-1.240272
C	10.546464	1.871369	-1.809869
N	11.212598	2.317888	-2.640948
O	9.96819	0.035499	3.813238
C	7.653902	-1.390044	-0.690151
C	5.359163	-1.744576	-0.709393
C	7.332347	-0.871323	0.545926
S	5.621795	-1.031533	0.846765
C	6.548166	-1.88211	-1.391699
H	6.608505	-2.309176	-2.385727
H	8.671633	-1.435977	-1.061469
F	15.747172	1.696246	-1.760969
F	17.004327	1.499905	0.558545

Table S9: Calculated energies (*E*) and energy gap (Δ*E*) of HOMO-1, LUMO+1, HOMO-2 and LUMO+2 for **PYFR** and **PYFD1-PYFD7**.

Compounds	HOMO-1	LUMO+1	Δ*E*	HOMO-2	LUMO+2	Δ*E*
**PYFR**	-6.290	-1.574	4.716	-6.742	-1.016	5.726
**PYFD1**	-6.270	-2.849	3.421	-6.564	-1.788	4.776
**PYFD2**	-6.350	-3.366	2.984	-6.656	-2.873	3.783
**PYFD3**	-6.352	-3.232	3.120	-6.658	-1.953	4.705
**PYFD4**	-6.346	-3.254	3.092	-6.648	-2.233	4.415
**PYFD5**	-6.329	-3.101	3.228	-6.625	-1.812	4.813
**PYFD6**	-6.303	-2.991	3.312	-6.594	-1.803	4.791
**PYFD7**	-6.287	-2.920	3.367	-6.584	-1.794	4.790

Table S10: Percentage contributions of donor, acceptor and π-spacer for HOMOs and LUMOs of **PYFR** and **PYFD1-PYFD7**.

Compounds	HOMOs			LUMOs		
	Donor	Acceptor	*π*-spacer	Donor	Acceptor	*π*-spacer
**PYFR**	83.4	-	16.6	61.3	-	38.7
**PYFD1**	83.1	0.1	16.9	0.0	92.1	7.9
**PYFD2**	83.7	0.1	16.2	0.0	80.8	19.2
**PYFD3**	83.8	0.1	16.1	0.0	91.1	8.9
**PYFD4**	83.6	0.1	16.3	0.0	91.6	8.4
**PYFD5**	83.5	0.1	16.5	0.0	90.7	9.3
**PYFD6**	83.2	0.1	16.8	0.0	91.6	8.4
**PYFD7**	83.3	0.1	16.6	0.0	92.3	7.7

Table S11: Natural bond orbitals (NBOs) analysis of **PYFR**.

Donor(*i*)	Type	Acceptor(*j*)	Type	*E*(2) [*kcal/mol*]	*E*(*j*)-*E*(*i*) [*a.u*]	*F*(*i,j*) [*a.u*]

C9-N37	σ	C25-C26	σ*	6.74	1.28	0.083
C9-C24	σ	N36-C60	σ*	6.07	1.09	0.073
C25-N37	σ	C8-C9	σ*	5.78	1.4	0.08
C3-C24	σ	C9-C24	σ*	5.69	1.28	0.076
C42-C44	σ	C44-C46	σ*	5.41	1.29	0.075
C62-C65	σ	C60-C62	σ*	4.12	1.3	0.065
C12-C13	σ	C14-C15	σ*	3.55	1.26	0.06
C9-C24	σ	C2-C3	σ*	3.1	1.28	0.056
C38-C39	σ	C29-C33	σ*	2.18	1.27	0.047
C28-H32	σ	C28-C31	σ*	1.11	1.12	0.032
C25-N37	σ	C25-N36	σ*	1.05	1.27	0.033
C2-H20	σ	C1-C2	σ*	1	1.11	0.03
C28-H32	σ	C26-C28	σ*	0.89	1.1	0.028
C56-H59	σ	C53-C56	σ*	0.7	1.1	0.025
C56-H59	σ	C55-C56	σ*	0.68	1.11	0.025
C25-N36	σ	C60-C61	σ*	0.63	1.39	0.026
C24-N36	σ	C9-N37	σ*	0.53	1.29	0.023
C47-H48	σ	C47-C51	σ*	0.51	0.95	0.02
C47-H49	σ	C47-C51	σ*	0.51	0.94	0.02
C24-N36	σ	C25-N37	σ*	0.5	1.35	0.023
C65-C67	π	C60-C62	π*	23.98	0.29	0.075
C65-C67	π	C60-C62	π*	23.98	0.29	0.075
C61-C63	π	C60-C62	π*	23.3	0.29	0.074
C42-C44	π	C38-C40	π*	23.28	0.29	0.074
C50-C51	π	C42-C44	π*	20.78	0.29	0.07
C8-C10	π	C9-C24	π*	19.76	0.29	0.068
C8-C10	π	C7-C14	π*	19.3	0.3	0.069
C2-C3	π	C9-C24	π*	18.81	0.3	0.068
C9-C24	π	C8-C10	π*	18.15	0.32	0.069
C9-C24	π	C25-N37	π*	16.11	0.29	0.062
C15-C16	π	C7-C14	π*	14.65	0.31	0.065
C4-C5	π	C15-C16	π*	14.22	0.3	0.063
C42-C44	π	C50-C51	π*	13.85	0.3	0.058
C60-C62	π	C28-C31	π*	0.60	0.32	0.012
N36	LP(1)	C25-N37	π*	51.64	0.3	0.111
N36	LP(1)	C9-C24	π*	34.74	0.32	0.095
N36	LP(1)	C60-C62	π*	1.64	0.3	0.02
N37	LP(1)	C25-N36	σ*	10.08	0.81	0.081
N37	LP(1)	C9-C24	σ*	6.93	0.96	0.074
N37	LP(1)	N36-C60	σ*	0.67	0.77	0.02

Table S12: Natural bond orbitals (NBOs) analysis of **PYFD1**.

**Donor(*i*)**	**Type**	**Acceptor(*j*)**	**Type**	***E*(2) [*kcal/mol*]**	***E*(*j*)-*E*(*i*) [*a.u*]**	***F*(*i,j*) [*a.u*]**
C69-H70	σ	C71-C72	σ*	9.4	1	0.086
C75-C91	σ	C91-N92	σ*	8.5	1.61	0.105
C91-N92	σ	C75-C91	σ*	8.34	1.57	0.103
C75-C89	σ	C89-N90	σ*	8.21	1.62	0.103
C89-N90	σ	C75-C89	σ*	8.11	1.57	0.101

C71-C73	σ	C76-O77	σ*	7.08	1.02	0.076
C9-N37	σ	C25-C26	σ*	6.59	1.28	0.082
C69-C71	σ	C71-C72	σ*	5.95	1.23	0.077
C5-C6	σ	C4-C5	σ*	4.77	1.27	0.07
C44-C50	σ	C42-C44	σ*	4.37	1.25	0.066
C94-C98	σ	C95-C98	σ*	3.84	1.28	0.063
C74-C78	σ	C73-C76	σ*	3.67	1.14	0.058
C78-C84	σ	O77-C80	σ*	2.56	1.04	0.046
C58-C60	σ	C60-H64	σ*	1.38	1.14	0.036
C82-H86	σ	C81-H83	σ*	0.74	0.93	0.023
C71-C72	σ	C76-O77	σ*	0.61	1.06	0.023
O77-C80	σ	C76-O77	σ*	0.53	1.3	0.024
C78-C84	σ	C74-C76	σ*	0.51	1.27	0.023
C78-C84	σ	C74-C76	σ*	0.51	1.27	0.023
C47-H49	σ	C47-C51	σ*	0.5	0.95	0.02
C81-C82	π	C78-C80	π*	25.5	0.28	0.078
C38-C39	π	C44-C46	π*	24.63	0.3	0.078
C63-C65	π	C58-C60	π*	24.09	0.29	0.075
C25-N37	π	C9-C24	π*	23.87	0.36	0.088
C7-C14	π	C3-C4	π*	22.89	0.28	0.072
C59-C61	π	C63-C65	π*	21.54	0.3	0.072
C1-C2	π	C5-C6	π*	20.9	0.3	0.072
C8-C10	π	C7-C14	π*	19.38	0.29	0.069
C74-C76	π	C78-C80	π*	14.4	0.32	0.063
C72-C75	π	C69-C71	π*	8.02	0.35	0.047
C73-O93	π	C74-C76	π*	4.64	0.43	0.043
C74-C76	π	C74-C76	π*	0.67	0.32	0.013
C91-N92	π	C89-N90	π*	0.65	0.48	0.016
C89-N90	π	C91-N92	π*	0.64	0.47	0.016
N36	LP(1)	C25-N37	π*	51.69	0.3	0.111
O77	LP(2)	C78-C80	π*	25.53	0.38	0.092
N36	LP(1)	C58-C60	π*	1.47	0.3	0.019
N90	LP(1)	C75-C89	σ*	12.69	1.04	0.103
N37	LP(1)	C25-N36	σ*	9.91	0.81	0.08
N37	LP(1)	N36-C58	σ*	0.68	0.77	0.021

Table S13: Natural bond orbitals (NBOs) analysis of **PYFD2**.

**Donor(*i*)**	**Type**	**Acceptor(*j*)**	**Type**	***E*(2) [*kcal/mol*]**	***E*(*j*)-*E*(*i*) [*a.u*]**	***F*(*i,j*) [*a.u*]**
C69-H70	σ	C71-C72	σ*	9.39	1	0.087
C75-C89	σ	C89-N90	σ*	8.59	1.62	0.106
C89-N90	σ	C75-C89	σ*	8.4	1.57	0.103
C78-C80	σ	C72-C74	σ*	7.86	1.18	0.086
C71-C73	σ	C76-O77	σ*	6.99	1.01	0.075
C69-C71	σ	C71-C72	σ*	6.14	1.24	0.078
C72-C75	σ	C75-C87	σ*	5.95	1.28	0.078
O77-C80	σ	C73-C76	σ*	5.38	1.39	0.078
C50-C55	σ	C44-C50	σ*	4.93	1.24	0.07
C29-H34	σ	C26-C27	σ*	4.4	1.09	0.062

C29-C33	σ	C27-C29	σ*	3.98	1.31	0.065
C60-H64	σ	C63-C65	σ*	3.64	1.11	0.057
C24-N36	σ	N36-C58	σ*	2.82	1.19	0.052
C25-C26	σ	C25-N36	σ*	1.76	1.11	0.04
C94-S95	σ	C92-C94	σ*	0.66	1.24	0.026
C84-C85	σ	C85-N99	σ*	0.63	1.02	0.023
N99-O101	σ	N99-O104	σ*	0.56	1.56	0.027
C47-H49	σ	C46-C47	σ*	0.51	0.94	0.02
C47-H49	σ	C47-C51	σ*	0.5	0.95	0.02
C81-C82	σ	C82-N100	σ*	0.5	1.02	0.021
C38-C39	π	C44-C46	π*	24.73	0.3	0.078
C31-C33	π	C26-C28	π*	24.48	0.29	0.076
C63-C65	π	C58-C60	π*	24.12	0.29	0.075
C55-C56	π	C52-C53	π*	20.16	0.3	0.07
C74-C76	π	C78-C80	π*	16.04	0.3	0.065
C72-C75	π	C74-C76	π*	9.36	0.32	0.05
N100-O102	π	N100-O102	π*	7.01	0.36	0.053
N99-O101	π	N99-O101	π*	6.59	0.4	0.054
C73-O91	π	C74-C76	π*	4.79	0.42	0.043
C72-C75	π	C72-C75	π*	1.51	0.33	0.02
C74-C76	π	C74-C76	π*	0.89	0.32	0.015
C87-N88	π	C89-N90	π*	0.61	0.47	0.015
C89-N90	π	C87-N88	π*	0.6	0.48	0.015
N36	LP(1)	C25-N37	π*	51.77	0.3	0.111
O103	LP(2)	C81-C82	π*	0.64	0.32	0.014
O91	LP(2)	C73-C76	σ*	24.22	0.73	0.12
O102	LP(2)	N100-O103	σ*	20.72	0.75	0.112
O102	LP(2)	C82-C85	σ*	0.56	0.84	0.02

Table S14: Natural bond orbitals (NBOs) analysis of **PYFD3**.

**Donor(*i*)**	**Type**	**Acceptor(*j*)**	**Type**	***E*(2) [*kcal/mol*]**	***E*(*j*)-*E*(*i*) [*a.u*]**	***F*(*i,j*) [*a.u*]**
C69-H70	σ	C71-C72	σ*	9.33	1	0.086
C75-C89	σ	C89-N90	σ*	8.63	1.61	0.106
C89-N90	σ	C75-C89	σ*	8.35	1.57	0.103
C78-C80	σ	C72-C74	σ*	7.81	1.19	0.086
C74-C76	σ	C78-C84	σ*	7.03	1.3	0.085
C9-N37	σ	C25-C26	σ*	6.56	1.28	0.082
C81-C82	σ	C82-C85	σ*	6.19	1.28	0.079
C71-C72	σ	C74-C78	σ*	6.07	1.19	0.076
C74-C78	σ	C78-C84	σ*	5.74	1.27	0.076
C42-C44	σ	C44-C46	σ*	5.5	1.29	0.075
C55-C56	σ	C56-C93	σ*	4.08	1.21	0.063
C44-C46	σ	C44-C50	σ*	3.18	1.2	0.055
C29-C33	σ	C38-C39	σ*	2.06	1.28	0.046
S99-O105	σ	C82-S99	σ*	1.01	0.92	0.028
C28-H32	σ	C31-H35	σ*	0.82	0.94	0.025
C65-H68	σ	C61-C65	σ*	0.71	1.11	0.025
C25-N36	σ	C58-C59	σ*	0.63	1.39	0.026

C47-H48	σ	C47-C51	σ*	0.51	0.95	0.02
C47-H49	σ	C47-C51	σ*	0.51	0.95	0.02
O77-C80	σ	C76-O77	σ*	0.51	1.31	0.023
C38-C39	π	C44-C46	π*	24.75	0.3	0.078
C31-C33	π	C26-C28	π*	24.5	0.29	0.076
C63-C65	π	C58-C60	π*	24.12	0.29	0.075
C50-C51	π	C55-C56	π*	24.02	0.28	0.074
C25-N37	π	C9-C24	π*	23.88	0.36	0.088
C8-C10	π	C9-C24	π*	19.84	0.29	0.068
C15-C16	π	C13-C14	π*	16.57	0.31	0.068
C92-C94	π	C93-C96	π*	15.78	0.31	0.063
C93-C96	π	C55-C56	π*	11.6	0.32	0.056
C73-O91	π	C74-C76	π*	4.76	0.42	0.043
C87-N88	π	C89-N90	π*	0.62	0.47	0.015
C89-N90	π	C87-N88	π*	0.59	0.48	0.015
N36	LP(1)	C25-N37	π*	51.75	0.3	0.111
N36	LP(1)	C9-C24	π*	34.53	0.32	0.095
O106	LP(2)	C81-C82	π*	0.75	0.32	0.014
O91	LP(2)	C71-C73	σ*	21.68	0.71	0.113
O105	LP(2)	C82-C85	σ*	0.53	0.9	0.02
C69-H70	σ	C71-C72	σ*	9.33	1	0.086

Table S15: Natural bond orbitals (NBOs) analysis of **PYFD4**.

**Donor(*i*)**	**Type**	**Acceptor(*j*)**	**Type**	***E*(2) [*kcal/mol*]**	***E*(*j*)-*E*(*i*) [*a.u*]**	***F*(*i,j*) [*a.u*]**
C69-H70	σ	C71-C72	σ*	9.44	1	0.087
C82-C101	σ	C101-N102	σ*	8.97	1.62	0.108
C85-C99	σ	C99-N100	σ*	8.84	1.62	0.107
C101-N102	σ	C82-C101	σ*	8.66	1.57	0.105
C75-C89	σ	C89-N90	σ*	8.58	1.62	0.106
C69-C71	σ	C71-C72	σ*	6.15	1.24	0.078
C9-C24	σ	N36-C58	σ*	6.05	1.09	0.073
C72-C75	σ	C75-C87	σ*	5.93	1.28	0.078
C69-C71	σ	C69-C94	σ*	5.71	1.31	0.077
C81-C82	σ	O77-C80	σ*	5.64	1.07	0.069
C92-H98	σ	C94-S95	σ*	5.52	0.75	0.057
C74-C78	σ	C78-C84	σ*	5.42	1.26	0.074
C84-C85	σ	C74-C78	σ*	5.29	1.23	0.072
C50-C51	σ	C50-C55	σ*	5.17	1.28	0.073
C39-H41	σ	C44-C46	σ*	4.94	1.1	0.066
C71-C73	σ	C73-O91	σ*	2.1	1.25	0.046
C71-C72	σ	C71-C73	σ*	1.02	1.12	0.03
C47-H48	σ	C47-C51	σ*	0.51	0.95	0.02
C47-H49	σ	C46-C47	σ*	0.51	0.94	0.02
C47-H49	σ	C47-C51	σ*	0.5	0.95	0.02
C38-C39	π	C44-C46	π*	24.72	0.3	0.078
C31-C33	π	C26-C28	π*	24.47	0.29	0.076
C63-C65	π	C58-C60	π*	24.12	0.29	0.075
C25-N37	π	C9-C24	π*	23.91	0.36	0.088

C74-C76	π	C72-C75	π*	22.49	0.32	0.076
C26-C28	π	C31-C33	π*	21.43	0.3	0.072
C55-C56	π	C52-C53	π*	20.15	0.3	0.07
C81-C82	π	C84-C85	π*	19.37	0.31	0.069
C84-C85	π	C78-C80	π*	18.54	0.29	0.067
C25-N37	π	C26-C28	π*	8.11	0.36	0.051
C87-N88	π	C89-N90	π*	0.61	0.47	0.015
C89-N90	π	C87-N88	π*	0.61	0.48	0.015
N36	LP(1)	C25-N37	π*	51.79	0.3	0.111
O77	LP(2)	C78-C80	π*	27.82	0.38	0.095
N36	LP(1)	C58-C60	π*	1.19	0.3	0.017
O91	LP(2)	C73-C76	σ*	24.11	0.73	0.12
N100	LP(1)	C85-C99	σ*	12.32	1.05	0.101
N37	LP(1)	N36-C58	σ*	0.68	0.77	0.021
C69-H70	σ	C71-C72	σ*	9.44	1	0.087

Table S16: Natural bond orbitals (NBOs) analysis of **PYFD5**.

**Donor(*i*)**	**Type**	**Acceptor(*j*)**	**Type**	***E*(2) [*kcal/mol*]**	***E*(*j*)-*E*(*i*) [*a.u*]**	***F*(*i,j*) [*a.u*]**
C69-H70	σ	C71-C72	σ*	9.48	1	0.087
C75-C89	σ	C89-N90	σ*	8.57	1.62	0.106
C89-N90	σ	C75-C89	σ*	8.39	1.57	0.103
C75-C87	σ	C87-N88	σ*	8.29	1.62	0.104
C78-C80	σ	C72-C74	σ*	7.96	1.18	0.087
C9-N37	σ	C25-C26	σ*	6.54	1.28	0.082
C71-C72	σ	C69-C71	σ*	6.11	1.3	0.08
C84-C85	σ	C82-C85	σ*	5.99	1.27	0.078
C81-C82	σ	O77-C80	σ*	5.56	1.07	0.069
C8-C10	σ	C7-C8	σ*	5.25	1.27	0.073
C40-C42	σ	C44-C50	σ*	5.00	1.23	0.07
C15-H18	σ	C5-C16	σ*	4.91	1.06	0.064
C55-H57	σ	C50-C51	σ*	4.84	1.1	0.065
C7-C14	σ	C7-C8	σ*	4.45	1.25	0.067
C44-C50	σ	C39-C46	σ*	3.92	1.27	0.063
C85-C99	σ	C81-C82	σ*	2.64	1.27	0.052
C25-N37	σ	C26-C28	σ*	1.89	1.42	0.046
C47-H49	σ	C46-C47	σ*	0.51	0.94	0.02
C69-H70	σ	C94-S95	σ*	0.51	0.73	0.017
C47-H49	σ	C47-C51	σ*	0.5	0.95	0.02
C38-C39	π	C44-C46	π*	24.7	0.3	0.078
C31-C33	π	C26-C28	π*	24.48	0.29	0.076
C63-C65	π	C58-C60	π*	24.12	0.29	0.075
C25-N37	π	C9-C24	π*	23.92	0.36	0.088
C52-C53	π	C50-C51	π*	22.96	0.31	0.076
C84-C85	π	C81-C82	π*	21.62	0.3	0.073
C78-C80	π	C81-C82	π*	20.77	0.3	0.072
C31-C33	π	C27-C29	π*	19.59	0.3	0.07
C26-C28	π	C25-N37	π*	18.94	0.28	0.065
C72-C75	π	C72-C75	π*	1.55	0.33	0.02

C87-N88	π	C89-N90	π*	0.61	0.47	0.015
C89-N90	π	C87-N88	π*	0.61	0.48	0.015
O77	LP(2)	C74-C76	π*	34.67	0.39	0.104
S95	LP(2)	C92-C94	π*	22.72	0.27	0.07
F101	LP(2)	C84-C85	π*	0.91	0.48	0.02
O91	LP(2)	C73-C76	σ*	23.86	0.73	0.119
O91	LP(2)	C71-C73	σ*	21.73	0.71	0.113
F104	LP(1)	C82-C100	σ*	0.5	1.46	0.024
C69-H70	σ	C71-C72	σ*	9.48	1	0.087

Table S17: Natural bond orbitals (NBOs) analysis of **PYFD6**.

**Donor(*i*)**	**Type**	**Acceptor(*j*)**	**Typ e**	***E*(2) [*kcal/mol*]**	***E*(*j*)-*E*(*i*) [*a.u*]**	***F*(*i,j*) [*a.u*]**
C69-H70	σ	C71-C72	σ*	9.5	1	0.087
C75-C89	σ	C89-N90	σ*	8.55	1.61	0.105
C89-N90	σ	C75-C89	σ*	8.37	1.57	0.103
C75-C87	σ	C87-N88	σ*	8.27	1.62	0.104
C87-N88	σ	C75-C87	σ*	8.19	1.57	0.102
C71-C73	σ	C76-O77	σ*	7.07	1.02	0.076
C74-C76	σ	C78-C84	σ*	6.79	1.29	0.084
C71-C72	σ	C69-C71	σ*	6.11	1.3	0.08
C78-C84	σ	C74-C78	σ*	5.82	1.23	0.075
C69-C71	σ	C69-C94	σ*	5.66	1.3	0.077
C92-C96	σ	C56-C93	σ*	5.19	1.21	0.071
C52-C53	σ	C47-C51	σ*	5.05	1.15	0.068
C40-C42	σ	C44-C50	σ*	5.00	1.23	0.07
C15-H18	σ	C7-C14	σ*	4.68	1.09	0.064
C10-H11	σ	C12-C13	σ*	3.82	1.11	0.058
C7-C8	σ	C4-C5	σ*	2.67	1.25	0.052
C44-C50	σ	C55-C56	σ*	1.85	1.22	0.043
C47-H49	σ	C46-C47	σ*	0.51	0.94	0.02
C47-H49	σ	C47-C51	σ*	0.51	0.95	0.02
C81-H83	σ	C82-Cl100	σ*	0.50	0.68	0.017
C38-C39	π	C44-C46	π*	24.67	0.3	0.078
C63-C65	π	C58-C60	π*	24.12	0.29	0.075
C25-N37	π	C9-C24	π*	23.91	0.36	0.088
C44-C46	π	C38-C39	π*	22.56	0.29	0.073
C59-C61	π	C63-C65	π*	21.58	0.3	0.072
C55-C56	π	C52-C53	π*	20.19	0.3	0.07
C69-C71	π	C72-C75	π*	19.48	0.3	0.07
C3-C4	π	C9-C24	π*	18.03	0.29	0.065
C15-C16	π	C5-C6	π*	16.52	0.31	0.068
C7-C14	π	C15-C16	π*	14.43	0.3	0.063
C74-C76	π	C74-C76	π*	0.72	0.32	0.014
C87-N88	π	C89-N90	π*	0.62	0.47	0.015
C89-N90	π	C87-N88	π*	0.62	0.48	0.015
N36	LP(1)	C25-N37	π*	51.78	0.3	0.111
S95	LP(2)	C93-C96	π*	27.18	0.28	0.078
N36	LP(1)	C58-C60	π*	1.20	0.3	0.017

O91	LP(2)	C73-C76	σ*	23.55	0.73	0.119
N88	LP(1)	C75-C87	σ*	12.7	1.04	0.103
Cl100	LP(2)	C80-C81	σ*	0.56	0.91	0.02

Table S18: Natural bond orbitals (NBOs) analysis of **PYFD7**.

**Donor(*i*)**	**Type**	**Acceptor(*j*)**	**Type**	***E*(2) [*kcal/mol*]**	***E*(*j*)-*E*(*i*) [*a.u*]**	***F*(*i,j*) [*a.u*]**
C69-H70	σ	C71-C72	σ*	9.35	1	0.086
C75-C89	σ	C89-N90	σ*	8.52	1.61	0.105
C89-N90	σ	C75-C89	σ*	8.34	1.57	0.103
C75-C87	σ	C87-N88	σ*	8.23	1.62	0.104
C71-C73	σ	C76-O77	σ*	7.14	1.02	0.076
C74-C76	σ	C78-C84	σ*	6.72	1.29	0.083
C96-H97	σ	C93-S95	σ*	5.65	0.75	0.058
C25-N36	σ	C3-C24	σ*	4.87	1.35	0.073
C44-C50	σ	C42-C44	σ*	4.37	1.25	0.066
C80-C81	σ	C82-F100	σ*	4.1	1.05	0.059
C94-S95	σ	C56-C93	σ*	3.99	1.18	0.061
C74-C76	σ	C74-C78	σ*	3.65	1.23	0.06
C5-C16	σ	C3-C4	σ*	3.29	1.23	0.057
C25-N36	σ	N36-C58	σ*	2.92	1.2	0.053
C10-C12	σ	C13-H23	σ*	2.58	1.13	0.048
C8-C9	σ	C10-C12	σ*	1.95	1.28	0.045
C4-C5	σ	C16-H17	σ*	1.78	1.1	0.04
C47-H49	σ	C46-C47	σ*	0.51	0.94	0.02
C78-C84	σ	C74-C76	σ*	0.51	1.28	0.023
C47-H49	σ	C47-C51	σ*	0.50	0.95	0.02
C38-C39	π	C44-C46	π*	24.65	0.3	0.078
C63-C65	π	C58-C60	π*	24.08	0.29	0.075
C25-N37	π	C9-C24	π*	23.84	0.36	0.088
C5-C6	π	C1-C2	π*	23.75	0.29	0.075
C69-C71	π	C73-O91	π*	22.06	0.31	0.074
C52-C53	π	C55-C56	π*	21.8	0.3	0.073
C74-C76	π	C73-O91	π*	20.89	0.33	0.075
C81-C82	π	C84-C85	π*	19.17	0.32	0.07
C84-C85	π	C78-C80	π*	18.62	0.3	0.07
C5-C6	π	C15-C16	π*	17.24	0.31	0.069
C89-N90	π	C87-N88	π*	0.64	0.48	0.016
C74-C76	π	C74-C76	π*	0.63	0.32	0.013
C81-C82	π	C81-C82	π*	0.55	0.31	0.012
C93-C96	π	C55-C56	π*	11.42	0.32	0.056
N36	LP(1)	C25-N37	π*	51.69	0.3	0.111
N36	LP(1)	C9-C24	π*	34.5	0.32	0.094
N36	LP(1)	C58-C60	π*	1.53	0.3	0.019
O91	LP(2)	C73-C76	σ*	23.46	0.74	0.119
N88	LP(1)	C75-C87	σ*	12.7	1.04	0.103
N37	LP(1)	N36-C58	σ*	0.68	0.77	0.021

Table S19: Wavelength, excitation energy and oscillator strength of investigated compound **PYFR**

NO	DFT λ (*nm*)	*E (eV)*	f_{os}	MO contributions
1	371.032	3.342	0.886	H→L (90%), H→L+4 (3%)
2	351.708	3.525	0.201	H→L+1 (75%), H-2→L (5%), H→L+2 (3%), H→L+4 (5%)
3	333.578	3.717	0.142	H-2→L+1 (14%), H→L+1 (13%), H→L+2 (29%), H→L+4 (13%), H-2→L (7%), H-1→L (6%), H→L+3 (3%), H→L+5 (5%), H→L+6 (4%)
4	323.626	3.831	0.127	H→L+2 (49%), H→L+3 (35%), H-2→L (2%), H-2→L+1 (2%), H-1→L (4%), H→L+4 (2%)
5	317.363	3.907	0.094	H→L+2 (13%), H→L+3 (53%), H→L+4 (18%), H-1→L (9%)

MO=molecular orbital, H=HOMO, L=LUMO, f_{os}= oscillator strength, wavelength= λ (*nm*)

Table S20: Wavelength, excitation energy and oscillator strength of investigated compound **PYFD1**

NO	DFT λ (*nm*)	*E (eV)*	f_{os}	MO contributions
1	553.649	2.239	0.013	H→L (97%)
2	501.554	2.472	0.005	H→L+1 (96%)
3	497.869	2.490	0.210	H-3→L (14%), H-2→L (25%), H-1→L (51%), H→L+1 (2%)
4	442.122	2.804	0.749	H-2→L+1 (18%), H-1→L+1 (56%), H-5→L (3%), H-3→L+1 (8%), H-1→L (7%)
5	421.328	2.943	0.197	H-3→L (22%), H-2→L (27%), H-1→L (36%), H-4→L (4%), H-3→L+1 (2%), H-2→L+1 (3%), H-1→L+1 (2%)
6	391.093	3.170	0.130	H-3→L+1 (23%), H-2→L+1 (26%), H-1→L+1 (36%), H-5→L (3%), H-4→L+1 (5%)

MO=molecular orbital, H=HOMO, L=LUMO, f_{os}= oscillator strength, wavelength= λ (*nm*)

Table S21: Wavelength, excitation energy and oscillator strength of investigated compound **PYFD2**

NO	DFT λ (*nm*)	*E (eV)*	f_{os}	MO contributions
1	685.981	1.807	0.008	H→L (99%)
2	663.053	1.870	0.002	H→L+1 (99%)
3	525.892	2.358	0.201	H-3→L (24%), H-3→L+1 (11%), H-1→L (40%), H-2→L (8%), H-2→L+1 (2%), H-1→L+1 (7%)
4	500.118	2.479	0.338	H-3→L+1 (15%), H-1→L (27%), H-1→L+1 (42%), H-2→L+1 (6%), H→L+2 (3%)
5	484.863	2.557	0.012	H→L+2 (97%)
6	468.041	2.649	0.253	H-3→L (35%), H-1→L (26%), H-1→L+1 (22%), H-4→L (9%), H-2→L (5%)

MO=molecular orbital, H=HOMO, L=LUMO, f_{os}= oscillator strength, wavelength= λ (*nm*)

Table S22: Wavelength, excitation energy and oscillator strength of investigated compound **PYFD3** at M06/6-311G(d,p) in gaseous phase.

NO	DFT λ (*nm*)	*E (eV)*	f_{os}	MO contributions
1	697.363	1.778	0.005	H→L (99%)
2	635.263	1.952	0.006	H→L+1 (99%)

3	533.105	2.326	0.216	H-3→L (34%), H-2→L (10%), H-1→L (48%), H-4→L (3%)
4	489.418	2.533	0.203	H-3→L (10%), H-3→L+1 (12%), H-1→L (20%), H-1→L+1 (43%), H-4→L (5%), H-2→L+1 (6%)
5	470.850	2.633	0.465	H-3→L (25%), H-1→L (29%), H-1→L+1 (25%), H-4→L (6%), H-3→L+1 (6%), H-2→L (4%), H-2→L+1 (3%)
6	443.656	2.795	0.013	H-2→L (84%), H-4→L (4%), H-3→L (9%)

MO=molecular orbital, H=HOMO, L=LUMO, f_{os}= oscillator strength, wavelength= λ (*nm*)

Table S23: Wavelength, excitation energy and oscillator strength of investigated compound **PYFD4** at M06/6-311G(d,p) in gaseous phase.

NO	DFT λ (*nm*)	*E (eV)*	f_{os}	MO contributions
1	671.419	1.847	0.007	H→L (97%), H→L+1 (2%)
2	640.150	1.937	0.005	H→L+1 (97%), H→L (2%)
3	522.897	2.371	0.183	H-3→L (35%), H-2→L (10%), H-1→L (44%), H-4→L (3%), H-3→L+1 (3%)
4	489.998	2.530	0.439	H-3→L+1 (15%), H-1→L (12%), H-1→L+1 (58%), H-2→L+1 (8%)
5	463.336	2.676	0.294	H-3→L (33%), H-1→L (40%), H-1→L+1 (10%), H-4→L (9%), H-2→L (4%)
6	443.895	2.793	0.495	H-3→L+1 (48%), H-2→L+1 (10%), H-1→L+1 (27%), H-4→L+1 (9%)

MO=molecular orbital, H=HOMO, L=LUMO, f_{os}= oscillator strength, wavelength= λ (*nm*)

Table S24: Wavelength, excitation energy and oscillator strength of investigated compound **PYFD5** at M06/6-311G(d,p) in gaseous phase.

NO	DFT λ (*nm*)	*E (eV)*	f_{os}	MO contributions
1	629.777	1.969	0.007	H→L (98%)
2	583.235	2.126	0.007	H→L+1 (98%)
3	512.310	2.420	0.213	H-3→L (35%), H-2→L (12%), H-1→L (44%), H-4→L (2%), H-3→L+1 (2%)
4	469.282	2.642	0.495	H-3→L+1 (15%), H-1→L (16%), H-1→L+1 (51%), H-3→L (3%), H-2→L+1 (8%)
5	447.693	2.769	0.412	H-3→L (33%), H-1→L (35%), H-1→L+1 (10%), H-4→L (6%), H-3→L+1 (5%), H-2→L (6%), H-2→L+1 (2%)
6	423.154	2.930	0.318	H-3→L+1 (44%), H-1→L+1 (34%), H-4→L+1 (8%), H-2→L+1 (8%)

MO=molecular orbital, H=HOMO, L=LUMO, f_{os}= oscillator strength, wavelength= λ (*nm*)

Table S25: Wavelength, excitation energy and oscillator strength of investigated compound **PYFD6** at M06/6-311G(d,p) in gaseous phase.

NO	DFT λ (*nm*)	*E (eV)*	f_{os}	MO contributions
1	591.923	2.095	0.009	H→L (98%)

2	540.943	2.292	0.011	H→L+1 (98%)
3	506.968	2.446	0.203	H-3→L (28%), H-2→L (18%), H-1→L (46%),
4	455.942	2.719	0.742	H-3→L+1 (14%), H-2→L+1 (12%), H-1→L (13%), H-1→L+1 (52%),
5	435.185	2.849	0.348	H-3→L (31%), H-2→L (12%), H-1→L (37%), H-4→L (5%), H-3→L+1 (5%), H-2→L+1 (3%), H-1→L+1 (5%)
6	407.588	3.042	0.207	H-3→L+1 (36%), H-2→L+1 (13%), H-1→L+1 (38%), H-4→L+1 (6%), H-1→L (2%)

MO=molecular orbital, H=HOMO, L=LUMO, f_{os}= oscillator strength, wavelength= λ (*nm*)

Table S26: Wavelength, excitation energy and oscillator strength of investigated compound **PYFD7** at M06/6-311G(d,p) in gaseous phase.

NO	DFT λ (*nm*)	*E (eV)*	f_{os}	MO contributions
1	589.082	2.105	0.008	H→L (98%)
2	529.191	2.343	0.012	H→L+1 (99%)
3	510.076	2.431	0.186	H-3→L (27%), H-2→L (18%), H-1→L (48%),
4	451.722	2.745	0.586	H-3→L+1 (13%), H-2→L+1 (11%), H-1→L (12%), H-1→L+1 (52%), H-5→L (2%), H-3→L (4%)
5	436.149	2.843	0.311	H-3→L (29%), H-2→L (12%), H-1→L (36%), H-4→L (5%), H-3→L+1 (6%), H-2→L+1 (3%), H-1→L+1 (7%)
6	403.253	3.075	0.176	H-3→L+1 (36%), H-2→L+1 (14%), H-1→L+1 (35%), H-5→L (2%), H-4→L+1 (6%)

MO=molecular orbital, H=HOMO, L=LUMO, f_{os}= oscillator strength, wavelength= λ (*nm*)

Table S27: Wavelength, excitation energy and oscillator strength of investigated compound **PYFR** at M06/6-311G(d,p) in chloroform solvent.

No	DFT λ (*nm*)	*E (eV)*	f_{os}	MO contributions
1	372.347	3.330	1.124	H→L (86%), H→L+1 (4%), H→L+2 (3%)
2	354.981	3.493	0.411	H→L+1 (79%), H-2→L (3%), H-2→L+2 (3%), H→L (6%), H→L+2 (6%)
3	333.443	3.718	0.283	H-2→L (14%), H-2→L+1 (11%), H-1→L (12%), H→L+2 (44%), H-1→L+1 (2%), H→L+1 (6%), H→L+5 (4%)
4	314.274	3.945	0.387	H-1→L (62%), H-1→L+1 (15%), H→L (2%), H→L+1 (4%), H→L+2 (8%)
5	300.197	4.130	0.022	H→L+3 (78%), H-2→L (2%), H→L+4 (6%), H→L+5 (5%)
6	297.160	4.172	0.031	H-1→L+1 (47%), H-3→L (9%), H-3→L+1 (4%), H-2→L (4%), H-1→L (7%), H→L+2 (6%), H→L+4 (8%), H→L+5 (5%)

MO=molecular orbital, H=HOMO, L=LUMO, f_{os}= oscillator strength, wavelength= λ (*nm*)

Table S28: Wavelength, excitation energy and oscillator strength of investigated compound **PYFD1** at M06/6-311G(d,p) in chloroform solvent.

No	DFT λ (*nm*)	*E (eV)*	f_{os}	MO contributions

1	528.807	2.345	0.142	H-2→L (10%), H-1→L (35%), H→L (53%),
2	506.906	2.446	0.082	H-2→L (19%), H-1→L (32%), H→L (46%),
3	470.136	2.637	0.250	H-1→L+1 (16%), H→L+1 (80%),
4	447.483	2.771	0.718	H-2→L+1 (20%), H-1→L+1 (54%), H→L+1 (19%), H-5→L (3%)
5	418.089	2.966	0.061	H-2→L (60%), H-1→L (29%), H-4→L (3%), H-3→L (2%), H-2→L+1 (3%)
6	385.044	3.220	0.048	H-2→L+1 (55%), H-1→L+1 (27%), H-5→L (8%), H-4→L+1 (3%), H-3→L+1 (2%)

MO=molecular orbital, H=HOMO, L=LUMO, f_{os}= oscillator strength, wavelength= λ (*nm*)

Table S29: Wavelength, excitation energy and oscillator strength of investigated compound **PYFD2** at M06/6-311G(d,p) in chloroform solvent.

No	DFT λ (*nm*)	*E (eV)*	f_{os}	MO contributions
1	583.592	2.125	0.059	H→L (89%), H-1→L (3%), H→L+1 (6%)
2	568.970	2.179	0.001	H→L+1 (89%), H-1→L+1 (3%), H→L (7%)
3	532.327	2.329	0.248	H-2→L (11%), H-2→L+1 (22%), H-1→L (22%), H-1→L+1 (33%), H-3→L+1 (2%), H→L+1 (4%)
4	500.542	2.477	0.778	H-2→L+1 (10%), H-1→L (48%), H-1→L+1 (22%), H-2→L (9%), H-1→L+2 (3%)
5	461.680	2.686	0.007	H→L+2 (95%), H-2→L (3%)
6	448.487	2.765	0.154	H-2→L (48%), H-1→L (20%), H-4→L (3%), H-3→L (6%), H-2→L+1 (6%), H-1→L+1 (9%), H→L+2 (3%)

MO=molecular orbital, H=HOMO, L=LUMO, f_{os}= oscillator strength, wavelength= λ (*nm*)

Table S30: Wavelength, excitation energy and oscillator strength of investigated compound **PYFD3** at M06/6-311G(d,p) in chloroform solvent.

No	DFT λ (*nm*)	*E (eV)*	f_{os}	MO contributions
1	582.195	2.130	0.043	H→L (92%), H-1→L (6%)
2	540.896	2.292	0.044	H→L+1 (95%), H-1→L+1 (3%)
3	536.125	2.313	0.237	H-2→L (32%), H-1→L (54%), H-3→L (3%), H→L (7%)
4	484.011	2.562	0.888	H-2→L+1 (22%), H-1→L+1 (67%), H→L+1 (3%)
5	448.860	2.762	0.108	H-2→L (47%), H-1→L (35%), H-4→L (3%), H-3→L (7%), H-2→L+1 (5%)
6	422.131	2.937	0.124	H-2→L+1 (54%), H-1→L+1 (27%), H-4→L+1 (4%), H-3→L+1 (7%), H-1→L (3%)

MO=molecular orbital, H=HOMO, L=LUMO, f_{os}= oscillator strength, wavelength= λ (*nm*)

Table S31: Wavelength, excitation energy and oscillator strength of investigated compound **PYFD4** at M06/6-311G(d,p) in chloroform solvent.

No	DFT λ (*nm*)	*E (eV)*	f_{os}	MO contributions
1	572.147	2.167	0.050	H→L (90%), H-1→L (6%), H→L+1 (2%)
2	545.825	2.272	0.041	H→L+1 (94%), H-1→L+1 (3%), H→L (2%)
3	529.666	2.341	0.210	H-2→L (32%), H-1→L (54%), H-3→L (3%), H→L (7%)

4	487.417	2.544	0.968	H-2→L+1 (22%), H-1→L+1 (68%), H→L+1 (3%)
5	443.133	2.798	0.111	H-2→L (47%), H-1→L (33%), H-4→L (3%), H-3→L (6%), H-2→L+1 (7%)
6	424.429	2.921	0.117	H-2→L+1 (54%), H-1→L+1 (26%), H-4→L+1 (3%), H-3→L+1 (6%), H-2→L (3%), H-1→L (5%)

MO=molecular orbital, H=HOMO, L=LUMO, f_{os}= oscillator strength, wavelength= λ (*nm*)

Table S32: Wavelength, excitation energy and oscillator strength of investigated compound **PYFD5** at M06/6-311G(d,p) in chloroform solvent.

**No**	**DFT λ (*nm*)**	***E (eV)***	f_{os}	**MO contributions**
1	557.408	2.224	0.063	H→L (88%), H-1→L (9%)
2	523.118	2.370	0.184	H-2→L (31%), H-1→L (52%), H→L (10%),
3	514.287	2.411	0.097	H→L+1 (93%), H-1→L+1 (5%)
4	470.243	2.637	0.926	H-2→L+1 (22%), H-1→L+1 (66%), H→L+1 (5%)
5	434.971	2.850	0.100	H-2→L (52%), H-1→L (33%), H-4→L (3%), H-3→L (5%), H-2→L+1 (5%)
6	408.084	3.038	0.099	H-2→L+1 (57%), H-1→L+1 (27%), H-4→L+1 (3%), H-3→L+1 (5%), H-1→L (3%)

MO=molecular orbital, H=HOMO, L=LUMO, f_{os}= oscillator strength, wavelength= λ (*nm*)

Table S33: Wavelength, excitation energy and oscillator strength of investigated compound **PYFD6** at M06/6-311G(d,p) in chloroform solvent.

**No**	**DFT λ (*nm*)**	***E (eV)***	f_{os}	**MO contributions**
1	544.818	2.276	0.090	H-1→L (17%), H→L (77%), H-2→L (4%)
2	518.893	2.389	0.141	H-2→L (27%), H-1→L (47%), H→L (22%),
3	494.178	2.509	0.149	H→L+1 (90%), H-1→L+1 (8%)
4	461.577	2.686	0.968	H-2→L+1 (22%), H-1→L+1 (62%), H-5→L (2%), H→L+1 (9%)
5	427.856	2.898	0.081	H-2→L (56%), H-1→L (31%), H-4→L (3%), H-3→L (3%), H-2→L+1 (4%)
6	397.998	3.115	0.089	H-2→L+1 (58%), H-1→L+1 (27%), H-5→L (2%), H-4→L+1 (3%), H-3→L+1 (4%)

MO=molecular orbital, H=HOMO, L=LUMO, f_{os}= oscillator strength, wavelength= λ (*nm*)

Table S34: Wavelength, excitation energy and oscillator strength of investigated compound **PYFD7** at M06/6-311G(d,p) in chloroform solvent.

**No**	**DFT λ (*nm*)**	***E (eV)***	f_{os}	**MO contributions**
1	541.345	2.290	0.104	H-1→L (24%), H→L (67%), H-2→L (7%)
2	517.463	2.396	0.115	H-2→L (24%), H-1→L (41%), H→L (32%),
3	481.174	2.577	0.174	H-1→L+1 (11%), H→L+1 (86%),
4	453.922	2.731	0.782	H-2→L+1 (21%), H-1→L+1 (59%), H→L+1 (13%), H-5→L (3%)
5	426.664	2.906	0.061	H-2→L (58%), H-1→L (31%), H-4→L (3%), H-3→L (3%), H-2→L+1 (3%)

6	391.327	3.168	0.069	H-2→L+1 (58%), H-1→L+1 (27%), H-5→L (4%), H-4→L+1 (3%), H-3→L+1 (3%)

MO=molecular orbital, H=HOMO, L=LUMO, f_{os}= oscillator strength, wavelength= λ (*nm*)

Table S35: Dipole polarizability and major contributing tensors (*D*) of the studied compounds (**PYFR** and **PYFD1-PYFD7**).

System	μ_x	μ_y	μ_z	μ_{total}
**PYFR**	-1.961	5.801	0.217	6.127
**PYFD1**	1.418	-9.884	0.732	10.013
**PYFD2**	-10.834	-10.973	4.495	16.062
**PYFD3**	-12.854	-10.894	5.677	17.780
**PYFD4**	-11.423	-10.697	5.028	16.438
**PYFD5**	7.040	10.473	3.533	13.105
**PYFD6**	-3.404	-10.368	2.892	11.290
**PYFD7**	1.908	10.480	2.011	10.841

Table S36: Average linear polarizability $\langle\alpha\rangle$ and major contributing tensor (*esu*) of the studied compounds (**PYFR** and **PYFD1-PYFD7**).

System	$\alpha_{xx}\times10^{-22}$	$\alpha_{yy}\times10^{-22}$	$\alpha_{zz}\times10^{-22}$	$\langle\alpha\rangle\times10^{-22}$
**PYFR**	1.589	1.187	4.380	1.072
**PYFD1**	2.723	1.420	1.108	1.750
**PYFD2**	2.961	1.445	1.176	1.861
**PYFD3**	2.998	1.482	1.217	1.899
**PYFD4**	3.019	1.426	1.170	1.872
**PYFD5**	2.874	1.429	1.152	1.819
**PYFD6**	2.925	1.417	1.160	1.834
**PYFD7**	2.732	1.417	1.160	1.752

Table S37: The computed first hyper-polarizability (β_{tot}) and major contributing tensors (*esu*) of (**PYFR** and **PYFD1-PYFD7**).

**First Hyper-polarizability**	**PYFR**	**PYFD1**	**PYFD2**	**PYFD3**	**PYFD4**	**PYFD5**	**PYFD6**	**PYFD7**
$\beta_{xxx}\times10^{-28}$	0.272	-1.437	-4.049	-3.328	-3.501	2.733	-1.749	1.568
$\beta_{xxy}\times10^{-29}$	0.096	-4.215	-9.629	-8.181	-9.345	7.201	-5.706	4.412
$\beta_{xyy}\times10^{-29}$	-1.101	-2.438	-3.593	-3.049	-3.738	2.892	-2.815	2.422
$\beta_{yyy}\times10^{-29}$	-0.243	-1.567	-1.616	-1.404	-1.704	1.408	-1.496	1.501
$\beta_{xxz}\times10^{-29}$	0.105	-4.968	-8.041	-7.396	-6.976	-6.020	-5.533	-5.652
$\beta_{yyz}\times10^{-30}$	-0.412	-5.263	-5.869	-5.131	-6.054	-5.139	-4.941	-5.185
$\beta_{xzz}\times10^{-29}$	0.0166	-1.552	-2.808	-2.596	-2.408	2.092	-1.672	1.786
$\beta_{yzz}\times10^{-30}$	-0.682	-3.784	-6.896	-5.611	-6.401	5.306	-4.544	4.201
$\beta_{zzz}\times10^{-30}$	0.0143	-7.662	-11.94	-13.80	-10.97	-11.55	-9.762	-9.078
$\beta_{tot}\times10^{-28}$	0.1650	2.036	4.937	4.128	4.365	3.445	2.431	2.204

Table S38: Second hyper-polarizability (γ_{tot}) and major contributing tensor (*esu*) of the studied compounds (**PYFR** and **PYFD7**).

System	$\gamma_x\times10^{-33}$	$\gamma_y\times10^{-34}$	$\gamma_z\times10^{-34}$	$\gamma_{tot}\times10^{-33}$
**PYFR**	1.025	0.4847	0.03492	1.077

**PYFD1**	2.574	2.278	1.185	2.921
**PYFD2**	4.180	2.735	1.772	4.631
**PYFD3**	3.609	2.432	1.647	4.017
**PYFD4**	3.841	2.717	1.572	4.269
**PYFD5**	3.157	2.294	1.429	3.529
**PYFD6**	3.095	2.383	1.338	3.467
**PYFD7**	2.664	2.264	1.279	3.018

Table S39: Frequency dependent First hyperpolarizability (*esu*) of studied comp. **PYFR** and **PYFD1-PYFD7**.

**Parameters**	**Frequency ω**	**PYFR** $\times 10^{-29}$	**PYFD1** $\times 10^{-28}$	**PYFD2** $\times 10^{-28}$	**PYFD3** $\times 10^{-28}$	**PYFD4** $\times 10^{-28}$	**PYFD5** $\times 10^{-28}$	**PYFD6** $\times 10^{-28}$	**PYFD7** $\times 10^{-28}$
$\beta\ (-\omega;\omega,0,)$	0.000	1.650	2.036	4.937	4.128	4.365	3.445	2.431	2.204
$\beta\ (\text{-}2,\ \omega;\omega,\omega)$	0.000	1.650	2.036	4.937	4.128	4.365	3.445	2.431	2.204
$\beta\ (-\omega;\omega,0)$	1907.21nm	1.823	2.118	5.048	4.234	4.453	3.502	2.544	2.282
$\beta\ (-2\omega;\omega,\omega)$	1907.21nm	2.388	2.849	6.904	5.769	6.021	4.682	3.503	3.101

Table S40: Frequency dependent Second hyperpolarizability (*esu*) of studied comp. **PYFR** and **PYFD1-PYFD7**.

**Parameters**	**Frequency ω**	**PYFR** $\times 10^{-33}$	**PYFD1** $\times 10^{-33}$	**PYFD2** $\times 10^{-33}$	**PYFD3** $\times 10^{-33}$	**PYFD4** $\times 10^{-33}$	**PYFD5** $\times 10^{-33}$	**PYFD6** $\times 10^{-33}$	**PYFD7** $\times 10^{-33}$
$\gamma(-\omega,\omega,0,0)$	0.000	1.077	2.921	4.632	4.017	4.269	3.529	3.467	3.018
	1907.21*nm*	1.008	2.929	4.883	4.185	4.430	3.618	3.516	3.041
$\gamma(-2\omega,\omega,\omega,0)$	0.000	1.077	2.921	4.632	4.017	4.269	3.529	3.467	3.018
	1907.21*nm*	1.139	3.779	7.539	6.114	6.430	4.998	4.686	3.992

Table S41: Calculated energies (E_{HOMO} , E_{LUMO}) and energy gap (ΔE) of the entitled compounds in eV at DFT approach.

**Compounds**	E_{HOMO}	E_{LUMO}	ΔE **(*eV*)**
**PYFR**	-5.735	-1.723	4.012
**PYFD1**	-5.729	-3.125	2.604
**PYFD2**	-5.739	-3.399	2.340
**PYFD3**	-5.740	-3.391	2.349
**PYFD4**	-5.738	-3.350	2.388
**PYFD5**	-5.735	-3.285	2.450
**PYFD6**	-5.731	-3.220	2.511
**PYFD7**	-5.733	-3.198	2.535

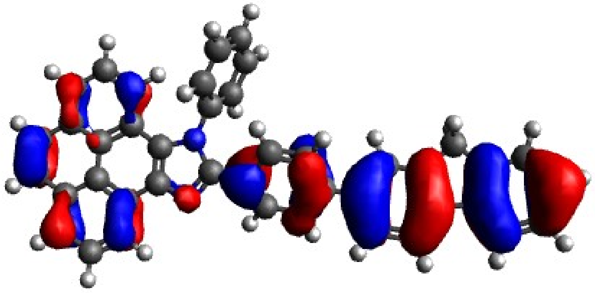

HOMO -1

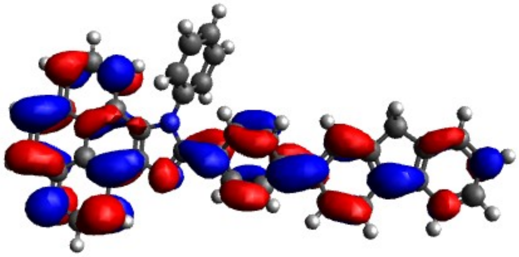

LUMO +1

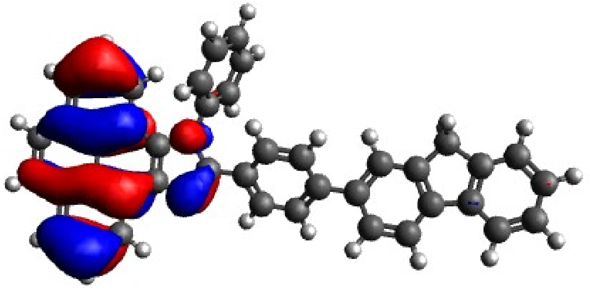

HOMO -2

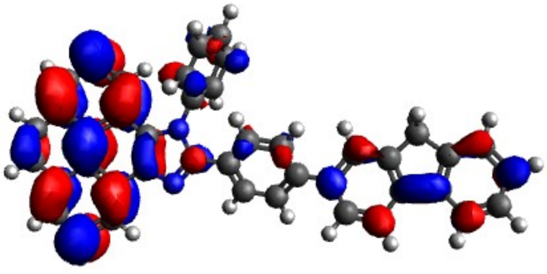

LUMO +2

PYFR

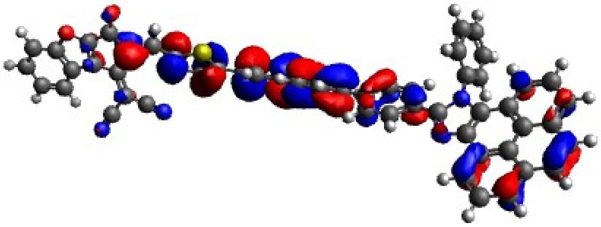

HOMO -1

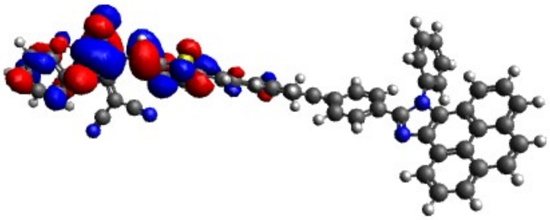

LUMO +1

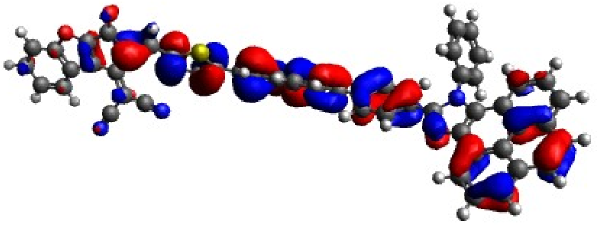

HOMO -2

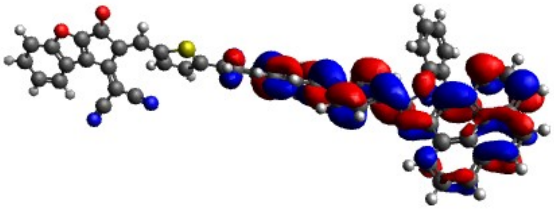

LUMO +2

PYFD1

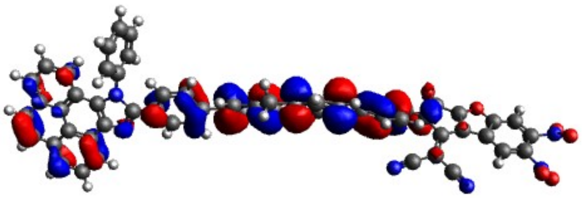

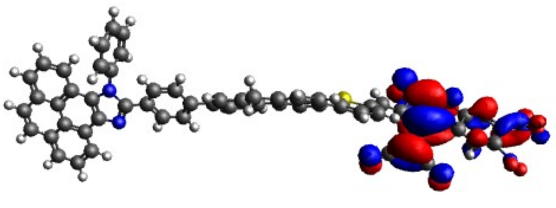

HOMO -1

LUMO +1

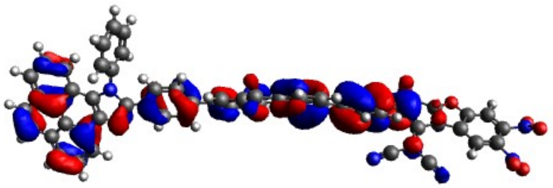

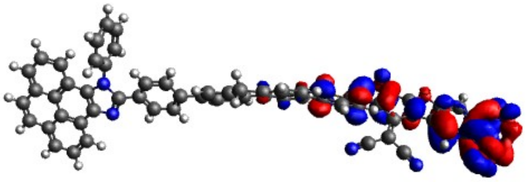

HOMO -2

LUMO +2

PYFD2

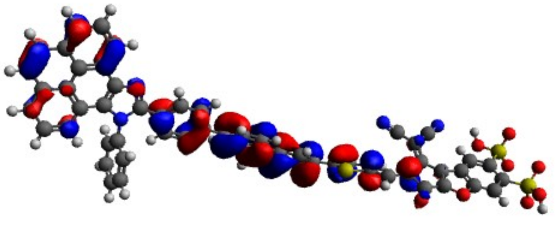

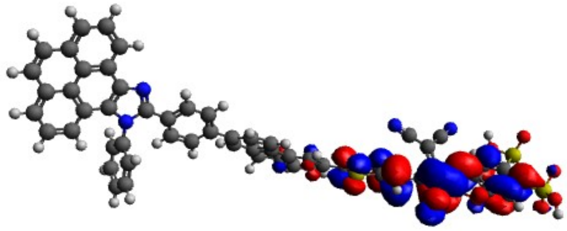

HOMO -1

LUMO +1

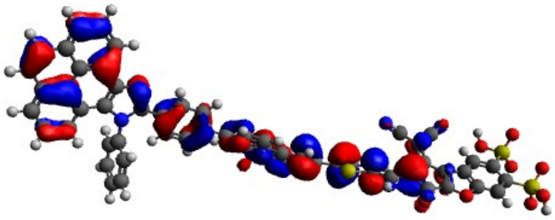

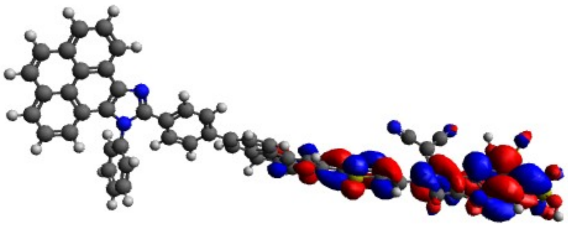

HOMO -2

LUMO +2

PYFD3

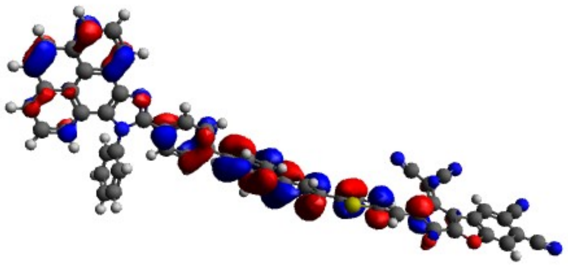

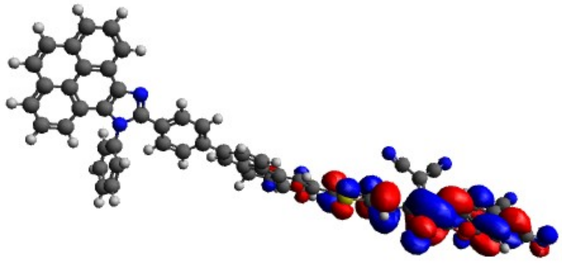

HOMO-1

LUMO+1

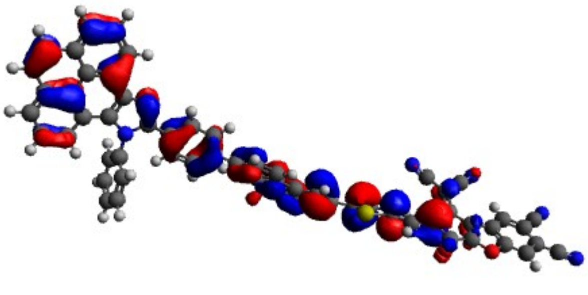

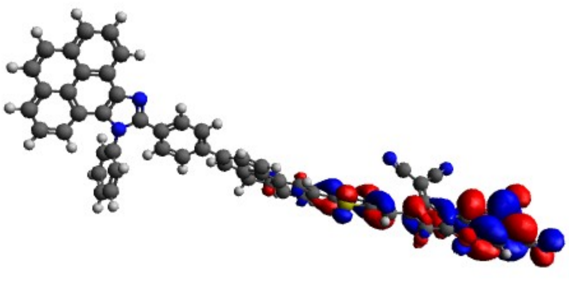

HOMO-2 **LUMO+2**

PYFD4

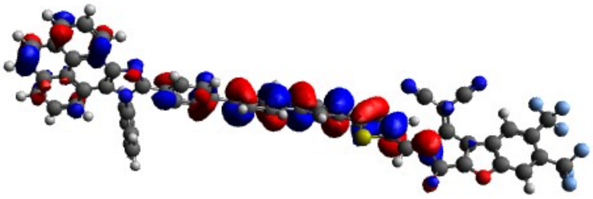

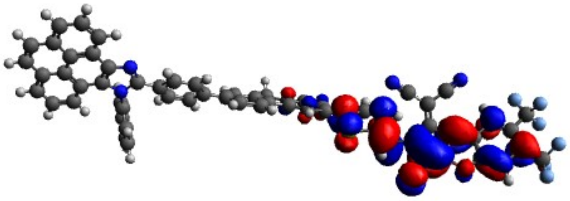

HOMO-1 **LUMO+1**

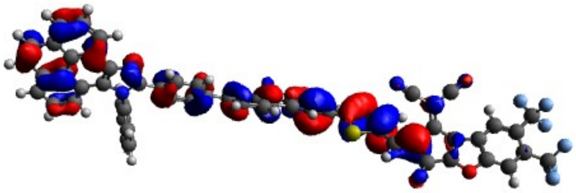

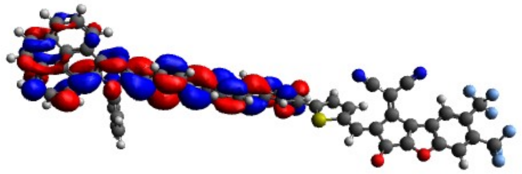

HOMO-2 **LUMO+2**

PYFD5

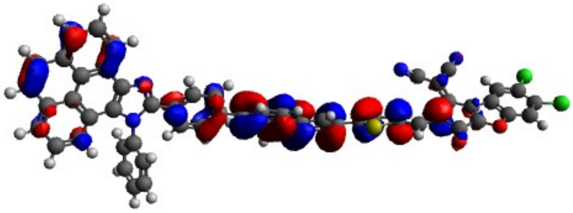

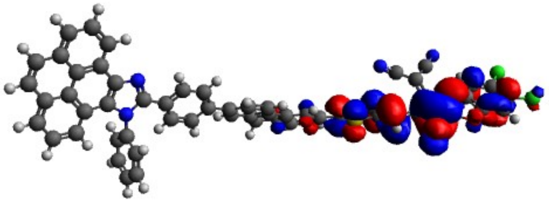

HOMO-1 **LUMO+1**

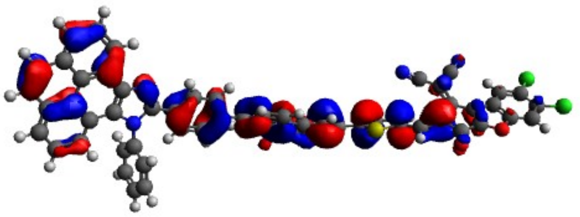

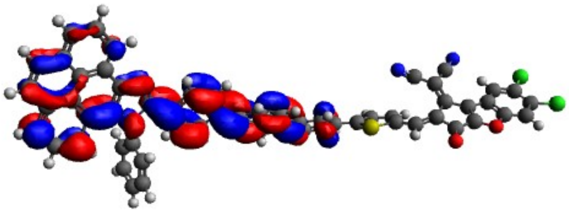

HOMO-2 **LUMO+2**

PYFD6

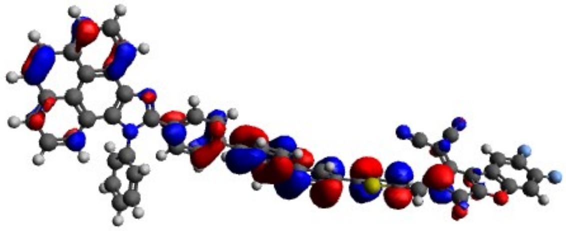

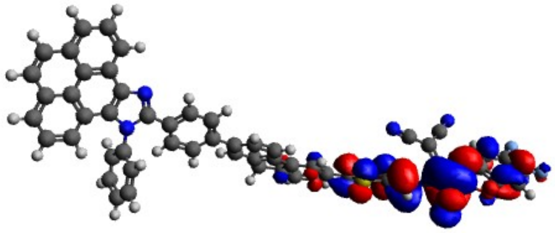

HOMO-1 **LUMO+1**

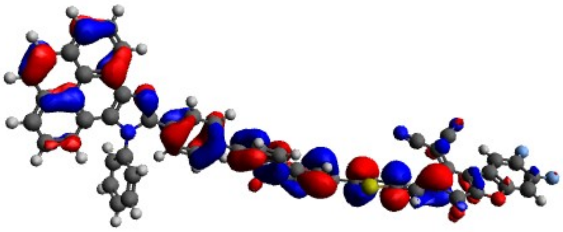

HOMO-2

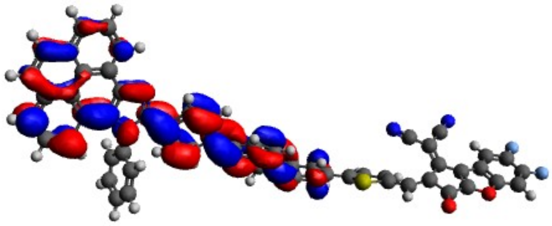

LUMO+2

PYFD7

Figure S1: HOMO-1/LUMO+1 and HOMO-2/LUMO+2 of **PYFR** and **PYFD1-PYFD7** compounds.
